# Supplementary material for: Structure-guided optimization of SLC1A1/EAAT3-selective inhibitors targeting renal cancer metabolism
Source: EMBO J. 2026 Apr 22;45(11):3763–87. doi: 10.1038/s44318-026-00776-2 (PMC13226657; doi:10.1038/s44318-026-00776-2)
Supplement: Supplementary file 1 — Appendix [file 44318_2026_776_MOESM1_ESM.pdf]

APPENDIX

for

Structure-Guided Optimization of SLC1A1/EAAT3-Selective Inhibitors Targeting Renal Cancer Metabolism

Table of Contents **Page No.**

Appendix Figure S1 02
Appendix Figure S2 04
Appendix Figure S3 06
Appendix Figure S4 08
Appendix Figure S5 10
Appendix Figure S6 13
Appendix Figure S7 15
Appendix Table S1 17
Appendix Table S2 19
Appendix Table S3 21

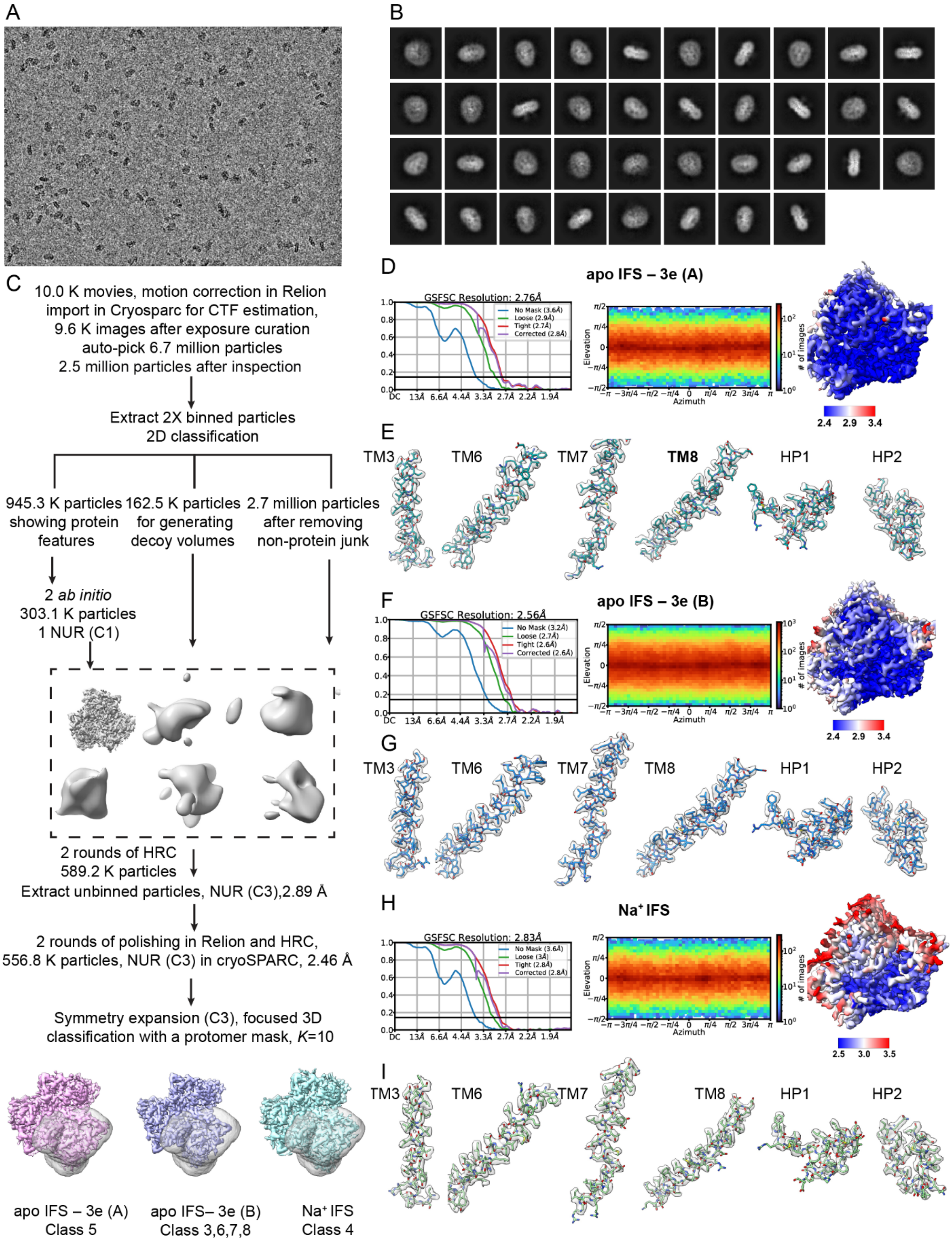
A
B
C
10.0 K movies, motion correction in Relion
import in Cryosparc for CTF estimation,
9.6 K images after exposure curation
auto-pick 6.7 million particles
2.5 million particles after inspection
Extract 2X binned particles
2D classification
945.3 K particles showing protein features
162.5 K particles for generating decoy volumes
2.7 million particles after removing non-protein junk
2 ab initio
303.1 K particles
1 NUR (C1)
2 rounds of HRC
589.2 K particles
Extract unbinned particles, NUR (C3),2.89 Å
2 rounds of polishing in Relion and HRC,
556.8 K particles, NUR (C3) in cryoSPARC, 2.46 Å
Symmetry expansion (C3), focused 3D
classification with a protomer mask, K=10
apo IFS – 3e (A)
Class 5
apo IFS– 3e (B)
Class 3,6,7,8
Na+ IFS
Class 4
D
apo IFS – 3e (A)
GSFSC Resolution: 2.76Å
No Mask (3.6Å)
Loose (2.9Å)
Tight (2.7Å)
Corrected (2.8Å)
Elevation
Azimuth
# of images
2.4 2.9 3.4
E
TM3
TM6
TM7
TM8
HP1
HP2
F
apo IFS – 3e (B)
GSFSC Resolution: 2.56Å
No Mask (3.2Å)
Loose (2.7Å)
Tight (2.6Å)
Corrected (2.6Å)
2.4 2.9 3.4
G
TM3
TM6
TM7
TM8
HP1
HP2
H
Na+ IFS
GSFSC Resolution: 2.83Å
No Mask (3.6Å)
Loose (3Å)
Tight (2.8Å)
Corrected (2.8Å)
2.5 3.0 3.5
I
TM3
TM6
TM7
TM8
HP1
HP2

19

Appendix Figure S1. Cryo-EM data processing and refinement. A representative image (**A**) and selected 2D class averages (**B**) of the dataset. (**C**) The cryo-EM data processing flow, showing identification of three protomer structural classes – two with bound 3e (classes A and B) and one bound to Na^+ ions. (**D-I**) The golden standard Fourier shell correlation (FSC) curves of the final refinement (**D, F, H, left**), the angular distribution of particles used for the final 3D reconstitutions (**D, F, H, middle**), the local resolution distribution (**D, F, H, right**), and the EM density of transport domain transmembrane helices (TMs), and helical hairpins (HPs) (**E, G, I**) for structural class A of *apo* IF state with **3e** (**D, E**), class B of *apo* IF state with **3e** (**F, G**), and Na^+-bound IF state (**H, I**). The map contour levels of (**E**), (**G**), and (**I**) in ChimeraX are 0.71, 0.732, and 0.654, respectively, corresponding to 5σ.

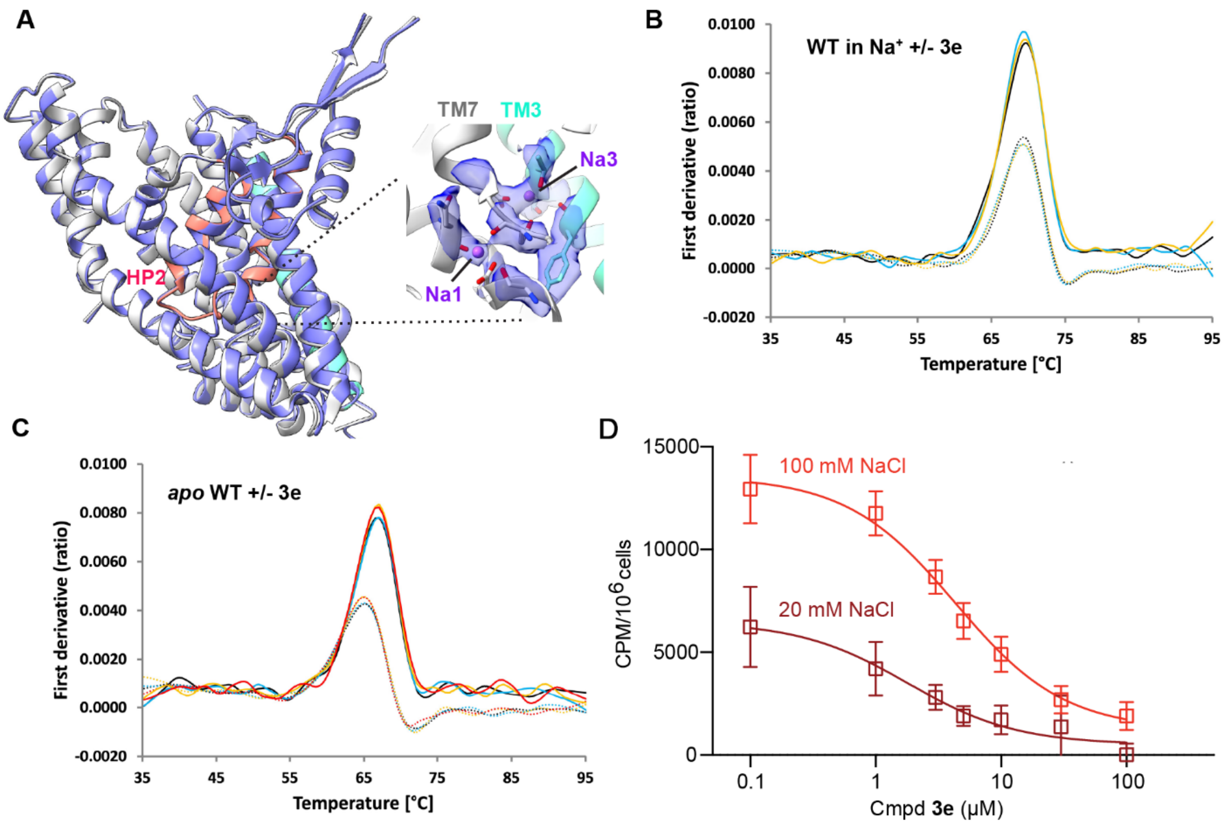

Appendix Figure S2. NanoDSF measuring WT SLC1A1 and compound 3e binding with and without Na⁺. (**A**) The Na⁺-bound monomer observed in the presence of **3e** and refined to 2.83 Å (gray with HP2 in pink and TM3 in cyan) is indistinguishable from Na⁺-bound SLC1A1g (state blue, PDB accession code 6x2l) imaged in the absence of **3e**; the entire protein was superposed. The inset shows the enlarged Na1 and Na3 sites with well-resolved EM density (contoured at 5 σ) for the bound Na⁺ ions. Coordinating residues are shown as sticks. (**B, C**) NanoDSF traces showing the first derivative of the fluorescence emission ratio at 350 and 330 nm of the WT SLC1A1g in 200 mM Na⁺ (**B**) or under *apo* conditions in buffer containing 100 mM NMDG (**C**) without (dashed lines) and with (solid lines) 100 μM **3e.** (**D**) L-[^{3}H]-Asp uptake into cells expressing SLC1A1g

in the presence of 100 (red) and 20 mM (brown) NaCl. Raw counts per 10^6 cells are shown; background counts in unresected cells averaged at ~1700±100 CPM were subtracted from the data. The fitted IC_{50} values are 4.3 ± 0.5 and 1.8 ± 0.7 μM, respectively.

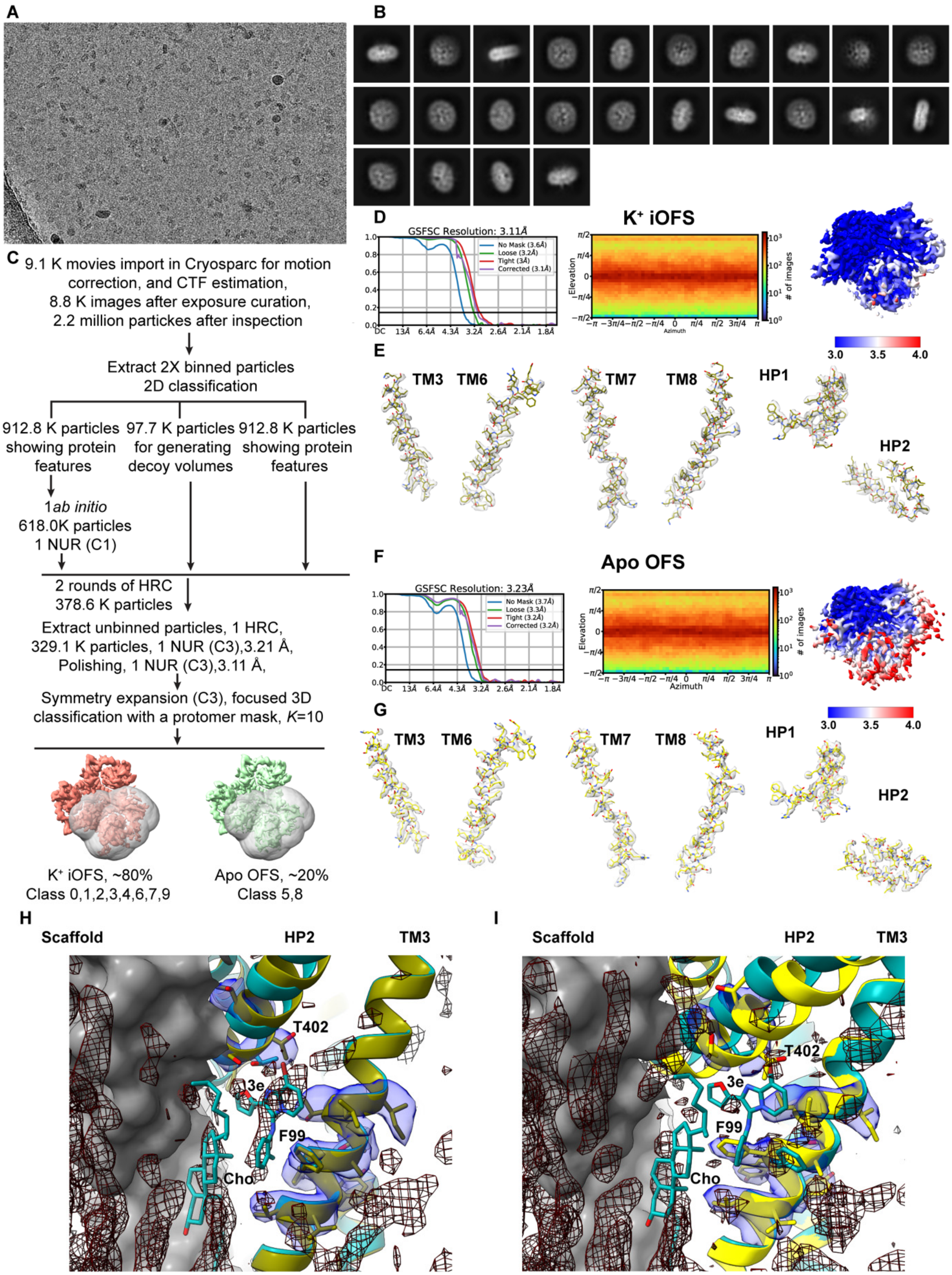
A
B
C
9.1 K movies import in Cryosparc for motion correction, and CTF estimation,
8.8 K images after exposure curation,
2.2 million partickes after inspection
Extract 2X binned particles
2D classification
912.8 K particles showing protein features
97.7 K particles for generating decoy volumes
912.8 K particles showing protein features
1*ab initio*
618.0K particles
1 NUR (C1)
2 rounds of HRC
378.6 K particles
Extract unbinned particles, 1 HRC,
329.1 K particles, 1 NUR (C3),3.21 Å,
Polishing, 1 NUR (C3),3.11 Å,
Symmetry expansion (C3), focused 3D classification with a protomer mask, *K*=10
K+ iOFS, ~80%
Class 0,1,2,3,4,6,7,9
Apo OFS, ~20%
Class 5,8
D
K+ iOFS
GSFSC Resolution: 3.11Å
No Mask (3.6Å)
Loose (3.2Å)
Tight (3Å)
Corrected (3.1Å)
Elevation
Azimuth
# of images
3.0
3.5
4.0
E
TM3
TM6
TM7
TM8
HP1
HP2
F
Apo OFS
GSFSC Resolution: 3.23Å
No Mask (3.7Å)
Loose (3.3Å)
Tight (3.2Å)
Corrected (3.2Å)
Elevation
Azimuth
# of images
G
TM3
TM6
TM7
TM8
HP1
HP2
3.0
3.5
4.0
H
Scaffold
HP2
TM3
T402
3e
F99
Cho
I
Scaffold
HP2
TM3
T402
3e
F99
Cho

Appendix Figure S3. Cryo-EM structure of Hg2+-crosslinked K269C/W441C SLC1A1g mutant in the presence of 100 µM 3e. A representative image (**A**) and selected 2D class averages (**B**). (**C**) The cryo-EM data processing flow, showing the identification of two protomer structural classes: an intermediate bound to a K^+ ion (K^+ iOFS) and the *apo* OF state (Apo OFS). (**D-G**) The golden standard Fourier shell correlation (FSC) curves of the final refinement (**D, F, left**), the angular distribution of particles used for the final 3D reconstitutions (**D, F, middle**), the local resolution distribution (**D, F, right**), and the EM density of transport domain transmembrane helices (TMs), and helical hairpins (HPs) (**E, G**) for K^+ iOFS state and Apo OFS. The map contour levels in ChimeraX are 0.098 (**E**) and 0.076 (**G**), corresponding to 4σ. (**H, I**) Molecular models (PDB IDs 8CUA, olive; and 8CUD, yellow) were rigid-body fitted without further adjustment into the density of K iOFS (3.11 Å resolution; **H**) and the Apo OFS (3.22 Å resolution; **I**. The transport domain from the **3e**-bound structure (teal) is superimposed for comparison using residues 84-124 and 275-475. TM6, HP1, and TM7 are omitted for clarity; residues F99, T402, compound **3e**, and cholesterol (Cho) are shown as sticks. The scaffold domain is displayed as a gray surface. Protein density in the vicinity of F99 and T402 is shown as a blue surface, whereas non-protein density is shown as a black mesh. No well-defined density corresponding to **3e** or cholesterol is observed, although a weak, elongated density is present at the **3e**-binding site in the K^+-bound intermediate state (**H**). Conformational shifts in HP2 disrupt interactions between T402 and **3e** and introduce steric clashes in the K^+-bound intermediate and *apo* OF transport domains, respectively. Map contour levels in ChimeraX are 1.23 (**H**) and 0.95 (**I**), corresponding to 5σ.

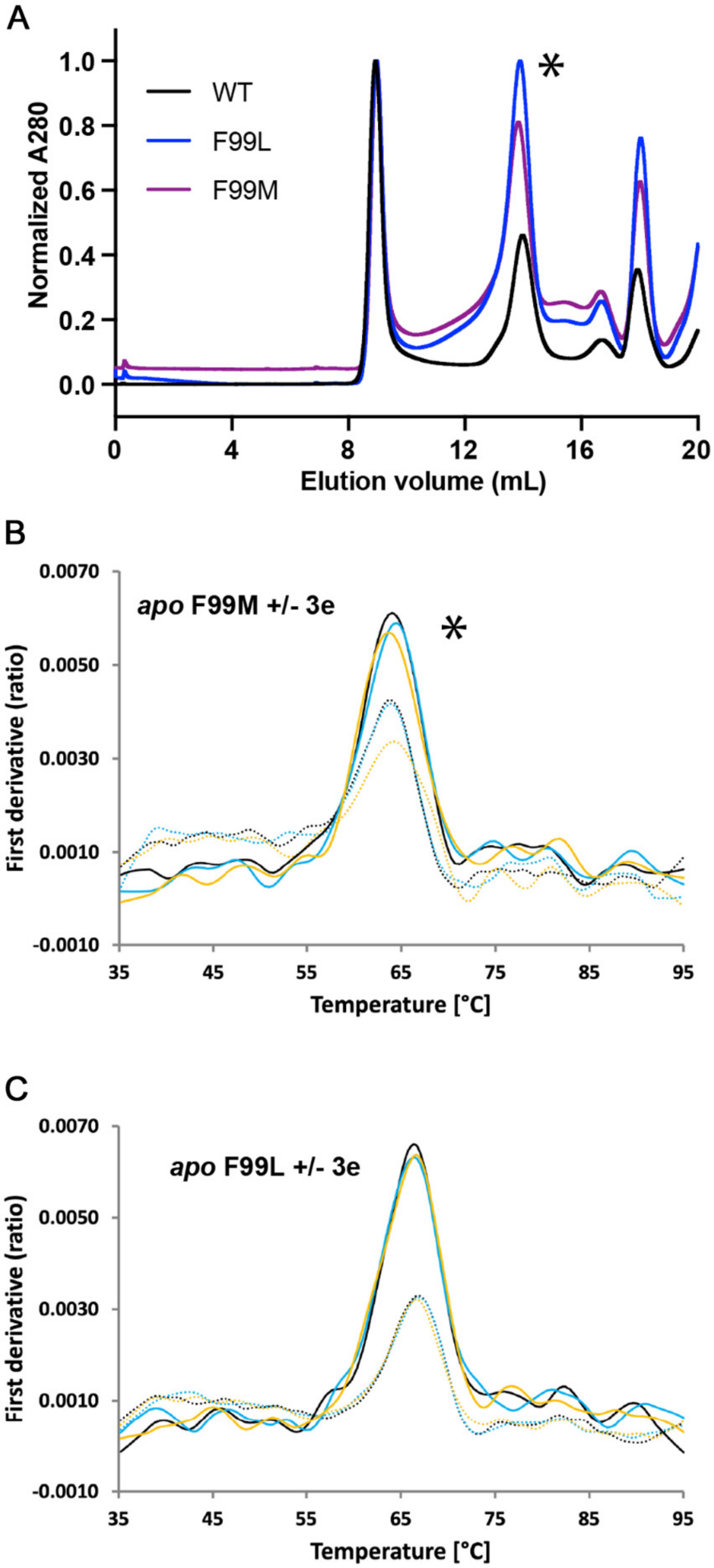
A
WT
F99L
F99M
*
Normalized A280
1.0
0.8
0.6
0.4
0.2
0.0
0
4
8
12
16
20
Elution volume (mL)
B
apo F99M +/- 3e
*
First derivative (ratio)
0.0070
0.0050
0.0030
0.0010
-0.0010
35
45
55
65
75
85
95
Temperature [°C]
C
apo F99L +/- 3e
First derivative (ratio)
0.0070
0.0050
0.0030
0.0010
-0.0010
35
45
55
65
75
85
95
Temperature [°C]

Appendix Figure S4. NanoDSF Measuring WT and mutant SLC1A1 purification and thermal stability. (**A**) Preparative size exclusion chromatography of the WT SLC1A1g and drug-resistant mutants F99L and F99M. An asterisk indicates the elution peak containing the trimeric protein, which was collected and used for nanoDSF experiments. (**B, C**) NanoDSF traces for the drug-resistant F99M (**B**) and F99L (**C**) mutants were recorded under *apo* conditions without (dashed lines) and with (solid lines) 100 μM **3e**. An asterisk indicates an experiment where two biological repeats exhibited slightly different absolute *Ti* values, while yielding similar ΔTi.

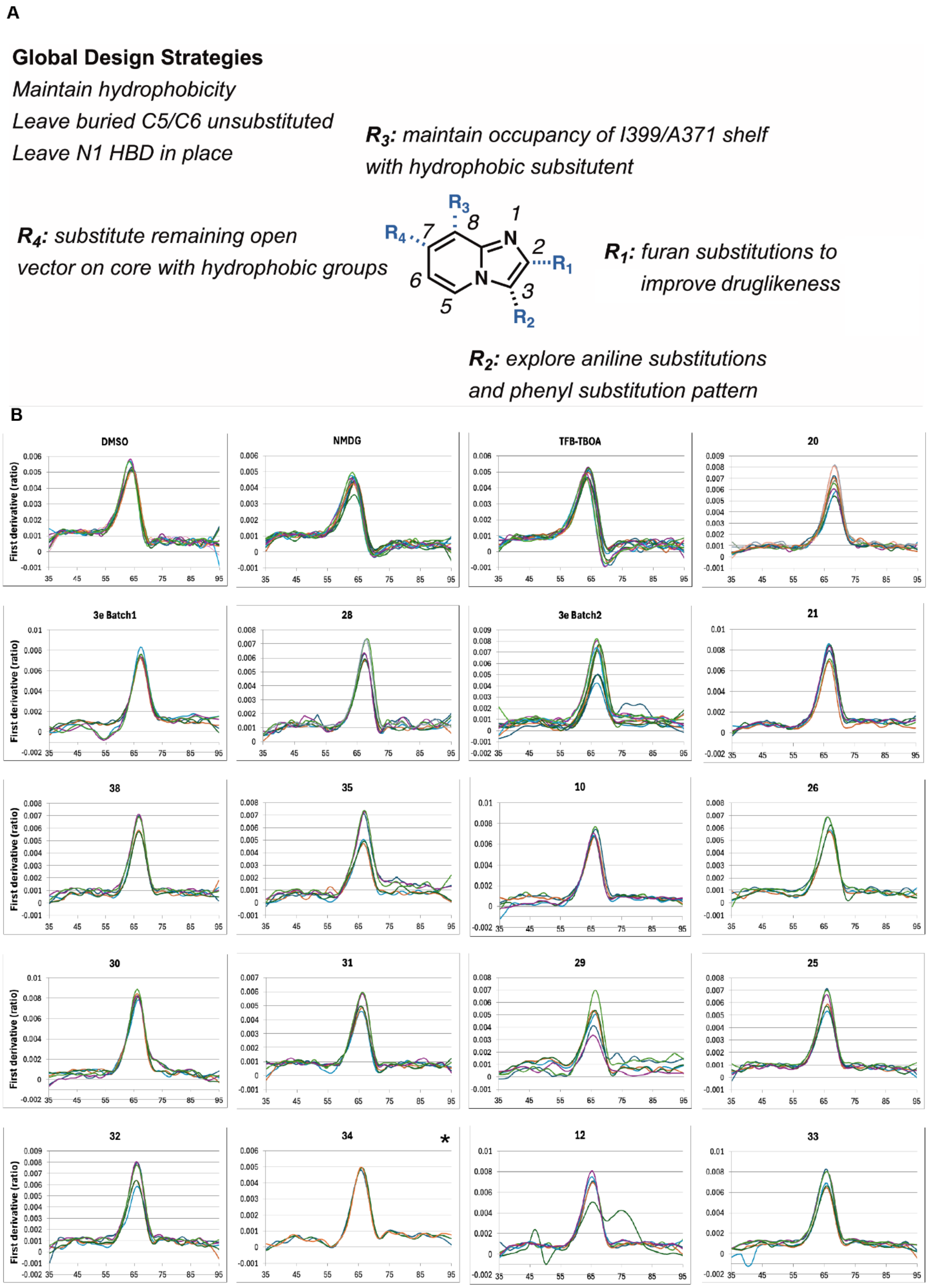
A
Global Design Strategies
Maintain hydrophobicity
Leave buried C5/C6 unsubstituted
Leave N1 HBD in place
R3: maintain occupancy of I399/A371 shelf with hydrophobic subsitutent
R4: substitute remaining open vector on core with hydrophobic groups
R1: furan substitutions to improve druglikeness
R2: explore aniline substitutions and phenyl substitution pattern
B
DMSO
NMDG
TFB-TBOA
20
3e Batch1
28
3e Batch2
21
38
35
10
26
30
31
29
25
32
34
*
12
33
First derivative (ratio)

B (continued)

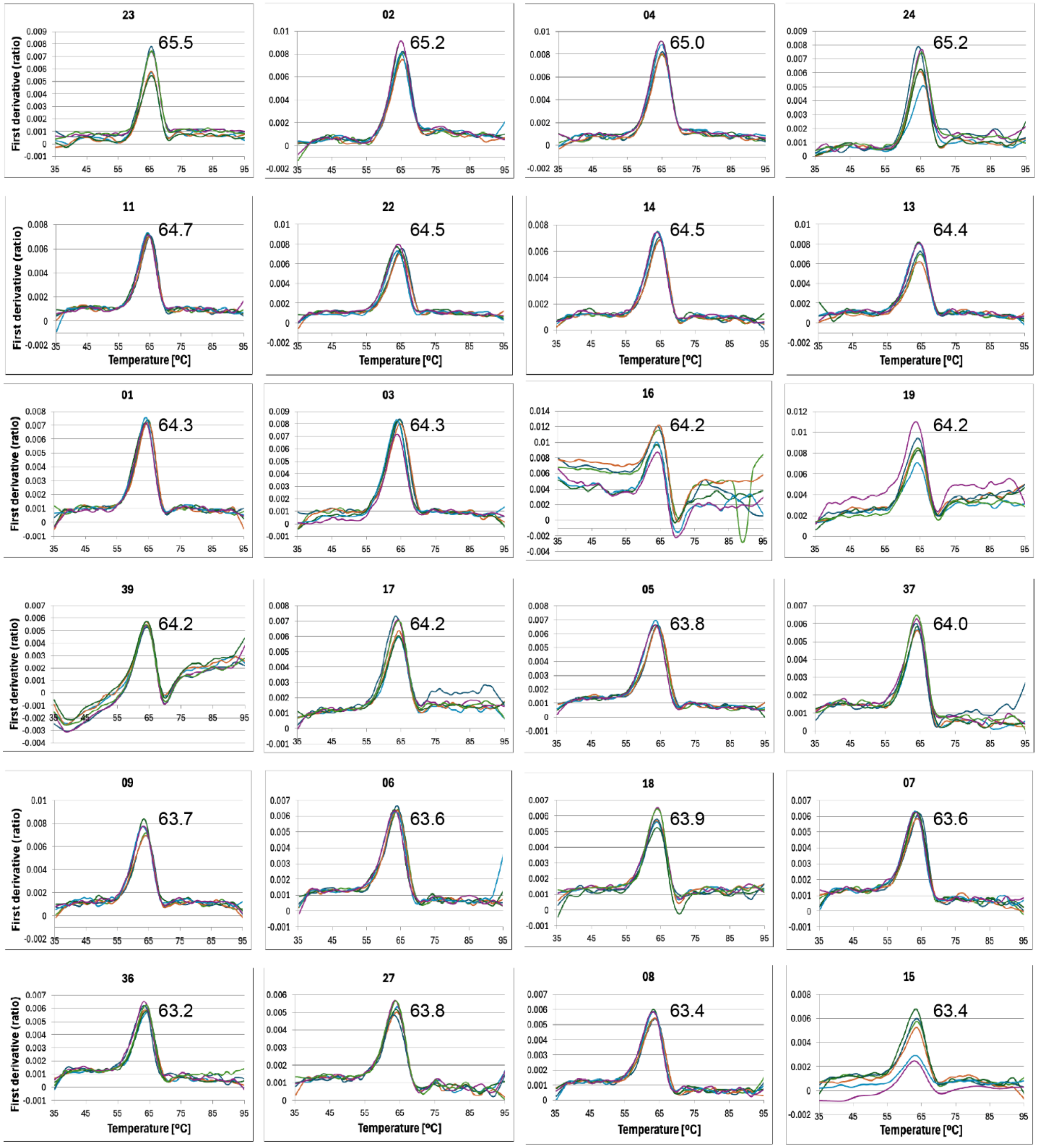

23
65.5
02
65.2
04
65.0
24
65.2
11
64.7
22
64.5
14
64.5
13
64.4
01
64.3
03
64.3
16
64.2
19
64.2
39
64.2
17
64.2
05
63.8
37
64.0
09
63.7
06
63.6
18
63.9
07
63.6
36
63.2
27
63.8
08
63.4
15
63.4
First derivative (ratio)
Temperature [ºC]

Appendix Figure S5. BIA analog design and raw nanoDSF data for BIA compound screening. (**A**) Optimization strategy of BIA analogs based on compound **3e**. (**B**) NanoDSF traces showing the first derivative of the fluorescence emission ratio at 350 and 330 nm of the SLC1A1g. The data were recorded under *apo* conditions in the presence of 200 mM NMDG chloride (NMDG) with additions of 10% DMSO or TFB-TBOA, which does not bind without Na^+ ions, as controls, and 100 µM of **3e** (two independently prepared batches) and its analogs, as indicated above the graphs. The inflection temperature (T_i) was determined from the maximum value of the peaks. The average T_i-s are shown on all plots. All measurements were performed using two independent protein preparations, each with at least three technical replicates. Each line in the graph represents an individual replicate. An asterisk (*) indicates a condition in which one biological replicate yielded an abnormal unfolding profile, excluded from the analysis.

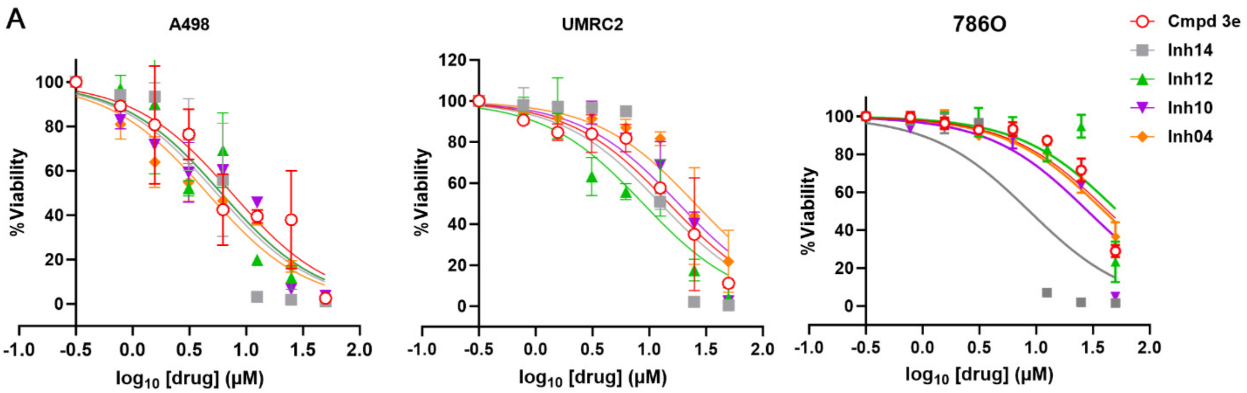

B

	Cmpd 3e	Inh14	Inh12	Inh10	Inh04
**A498**	7.441	5.464	6.033	5.998	4.527
**UMRC2**	14.92	12.57	8.847	18.15	25.39
**786O**	43.07	8.74	52.48	28.55	39.73

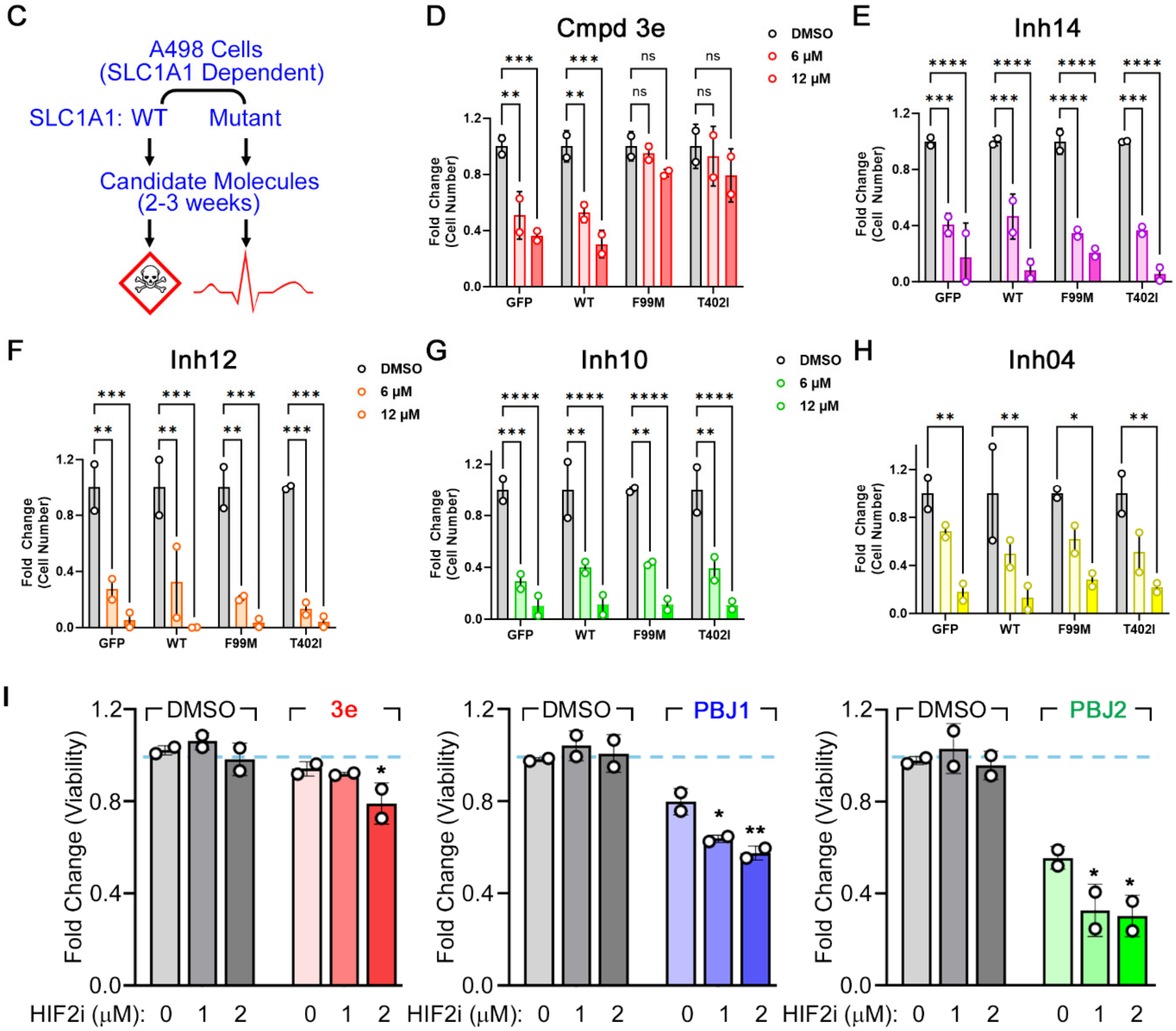

Appendix Figure S6. On-target validation of candidate BIA analogs in cell-based assays. (**A** and **B**) Cell viability measurement of the indicated RCC cells (**A**) and a table with the IC_{50} values, calculated using regression analysis in Graphpad Prism (**B**), upon treatment with the indicated compounds for 7 days. (**C**) Schema depicting the experimental design to establish on-target effects of cmpd **3e** analogs using the SLC1A1 mutants. (**D** to **H**) Fold change in cell number, plotted relative to cell counts in the DMSO-treated arm, of A498 cells expressing the indicated SLC1A1 constructs or GFP control, treated with the indicated concentrations of **3e** (**D**), **14** (**E**), **12 (F**), **10** (**G**), and **04** (**H**), for 21 days. Cell counts, measured using ViCell, were compared using ANOVA (relative to GFP within each group) with Dunnett's multiple comparison test, n=2, *p<0.05, **p<0.01, ***p<0.001, ****p<0.0001, ns=non-significant. (**I**) Fold change in cell counts, calculated after 7 days of exposure, in A498 cells that were treated with either 12 µM of the indicated SLC1A1 inhibitor or DMSO (control), in the presence or absence of the indicated concentrations of the HIF2α inhibitor PT2385.

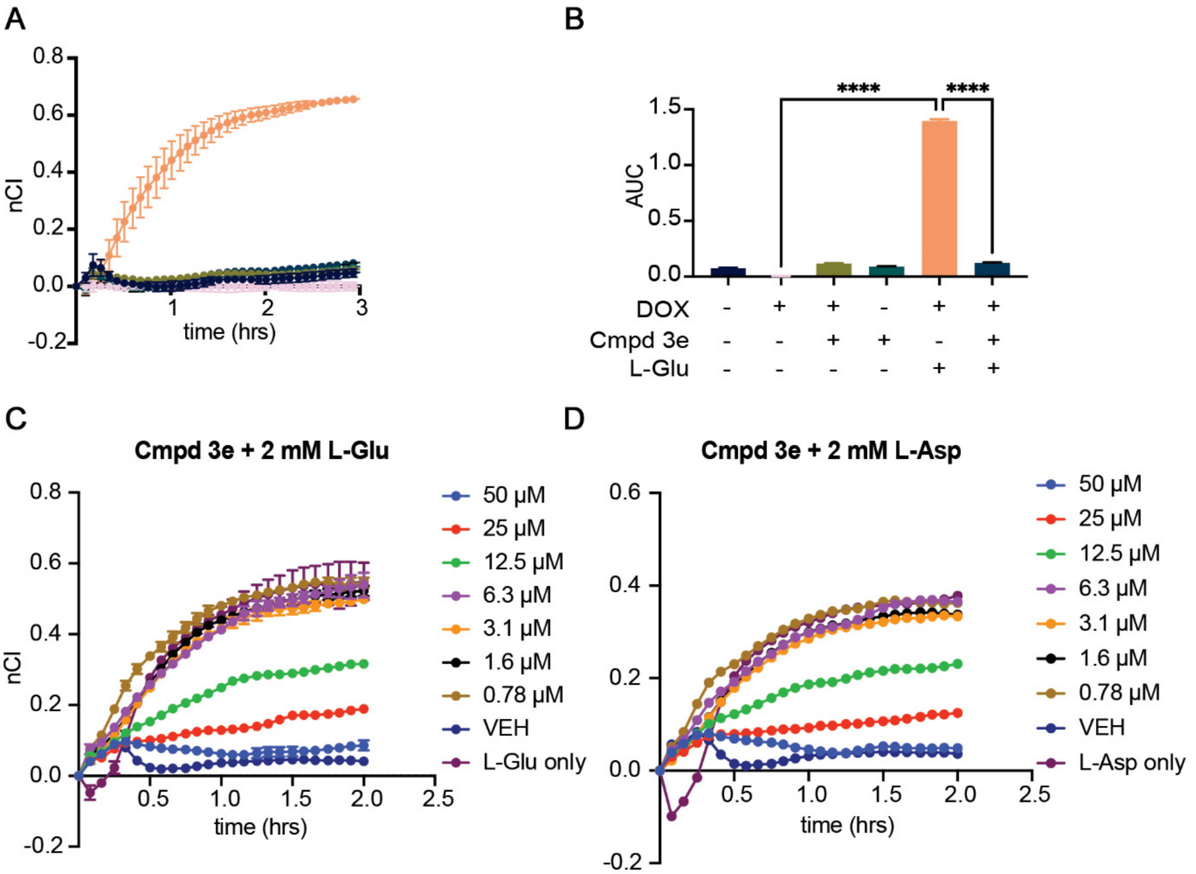

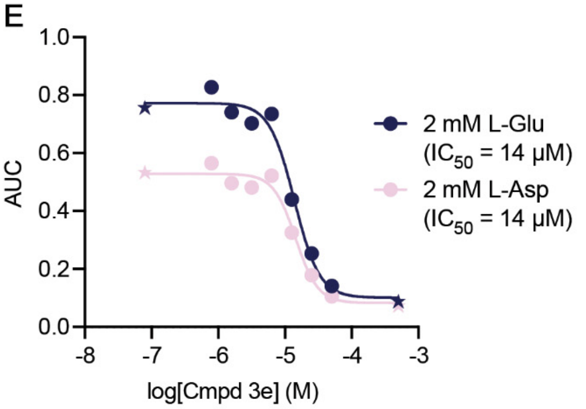

F

	SLC1A1	SLC1A2	SLC1A3
TFB-TBOA	8 ± 0	0.028 ± 0.004	0.10 ± 0.02
**3e**	10 ± 3	> 50	> 50
PBJ1	9 ± 1	23 ± 1	> 50
PBJ2	12 ± 3	> 50	> 50

G

Membrane Potential Live Cell Kinetic Assay

	Cmpd 3e	PBJ1	PBJ2
Bottom	1.413e-06	6.561e-06	1.248e-06
Top	96.68	89.38	97.69
LogEC50	-5.695	-5.310	-5.900
HillSlope	-1.705	-1.264	-1.671
EC50	2.018e-06	4.897e-06	1.258e-06
Span	96.68	89.38	97.69

Appendix Figure S7. Compound 3e inhibits SLC1A1-mediated uptake of both L-Glu and L-Asp. (A-B) Normalized cell index (nCI) (**A**) and integrated Are Under the Curve (AUC) (**B**) for HEK293-SLC1A1 cells treated with 0.15 µg/mL doxycycline (DOX), 1 mM L-Glu, and/or 50 µM **3e** during an xCELLigence impedance assay. HEK293-SLC1A1 cells respond to L-Glu with an increase in nCI when SLC1A1 expression is induced with Dox (orange), but the cells do not respond to L-Glu when Dox is omitted (green). The addition of **3e** inhibits the Dox-dependent morphological response to L-Glu (navy). (**C**-**E**) Impedance based measurement of nCI (**C** and **D**) or integrated AUC (**E**) in HEK293-SLC1A1 cells that were treated with the indicated doses of cmpd **3e**, or vehicle (VEH) control, either in the presence of 2 mM L-Glu or 2 mM L-Asp, as indicated. Data are presented as mean ± S.D. from two technical replicates. (**F**) IC_{50} values measured from the impedance assays, described in main figure 6D. (**G**) IC_{50} values measured from the membrane potential FLIPR assays, described in main figures 6E and 6F.

Appendix Table S1. Cryo-EM data collection, refinement, and validation statistics

	apo IFS - **3e** (A)	*apo* IFS - **3e** (B)	Na^+ IFS	K^+ iOFS	Apo OFS
**Data collection and processing**					
Magnification		100,500 X		81,000 X	
Voltage (kV)		300		300	
Electron exposure (e–/Å^2)		58		53.9	
Defocus range (μm)		-1.0 – -2.0		-0.8 – -2.5	
Pixel size (Å)		0.83		0.856	
Initial particle images (no.)		2,717,842 (1,470,412, C3 expanded particles)		2,187,170 (987,213)	
Symmetry imposed	C1	C1	C1	C1	C1
Final particle images (no.)	165,757	659,564	165,684	791,674	195,539
Map resolution (Å)	2.76	2.56	2.83	3.11	3.23
FSC threshold	0.143	0.143	0.143	0.143	0.143
Map resolution range (Å)	39.45 – 2.43	32.54 – 1.81	40.85 – 2.47	37.15 – 1.83	40.87 – 2.64
**Refinement**					
Initial model used (PDB code)	6X3F	6X3F	6X2L	This map is similar to EMD-26997. The model 8CUA can be fitted in this map.	This map is similar to EMD-26998. The model 8CUD can be fitted in this map.
Model resolution (Å)	2.9	2.7	3.1		
FSC threshold	0.5	0.5	0.5		
Map sharpening *B* factor (Å^2)					
Model composition					
Non-hydrogen atoms	3,224	3,238	3,167		
Protein residues	412	409	407		
Ligands	3	4	4		
B factors (Å^2)					
Protein	46.66	35.81	47.25		
Ligand	52.58	40.91	48.34		
R.m.s. deviations					
Bond lengths (Å)	0.005	0.005	0.004		

Bond angles (°)	0.949	1.007	0.928		
Validation					
MolProbity score	1.23	1.02	1.21		
Clashscore	4.51	2.39	4.27		
Poor rotamers (%)	0.00	0.00	0.00		
Ramachandran plot					
Favored (%)	98.03	99.01	98.25		
Allowed (%)	1.97	0.99	1.75		
Disallowed (%)	0.00	0.00	0.00		
**PDB code**	9P4X	9P4Y	9P4Z		
**EMDB code**	71288	71289	71290	75048	75049

Appendix Table S2. Structure activity relationship (a)

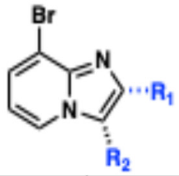

	R1	R2	NanoDSF ΔT_i (°C)	Impedance IC_{50} (µM)	384w CTG® GI_{50} (µM)
**3e**	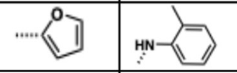		+ 3.1 (n = 12)	9.7 (n = 9)	42 (n = 8)
**1**	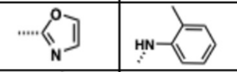		+ 0.5 (n = 6)	n.d.	45 (n =2)
**2**	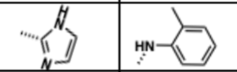		+ 1.4 (n = 6)	n.d.	40 (n = 2)
**3**	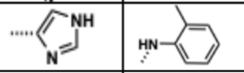		+ 0.5 (n = 6)	n.d.	29 (n = 2)
**4**	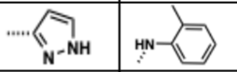		+ 1.1 (n = 6)	n.d.	23 (n = 2)
**5**	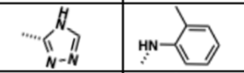		- 0.1 (n = 6)	n.d.	> 50 (n = 3)
**6**	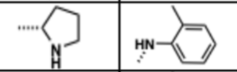		- 0.2 (n = 6)	n.d.	26 (n = 2)
**7**	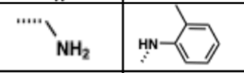		- 0.2 (n = 6)	n.d.	36 (n = 2)
**8**	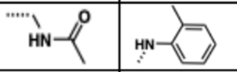		- 0.5 (n = 6)	n.d.	n.d.
**9**	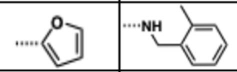		- 0.2 (n = 6)	n.d.	> 50 (n = 3)
**10**	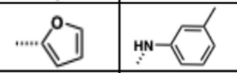		+ 2.2 (n = 6)	n.d.	12 (n = 5)
**11**	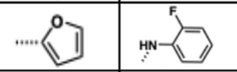		+ 0.8 (n = 6)	n.d.	> 50 (n = 2)
**12**	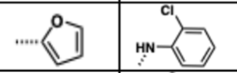		+ 1.7 (n = 6)	n.d.	12 (n = 5)
**13**	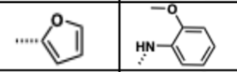		+ 0.5 (n = 6)	n.d.	40 (n = 2)
**14**	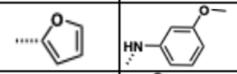		+ 0.6 (n = 6)	n.d.	1.8 (n = 5)
**15**	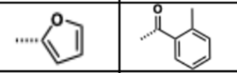		- 0.5 (n = 6)	n.d.	> 50 (n = 2)
**16**	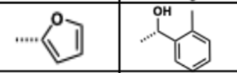		+ 0.3 (n = 6)	n.d.	> 50 (n = 2)
**17**	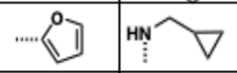		+ 0.0 (n = 6)	n.d.	> 50 (n = 2)
**18**	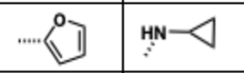		- 0.2 (n = 6)	n.d.	> 50 (n = 2)
**19**	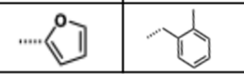		+ 0.1 (n = 6)	n.d.	44 (n = 2)

Appendix Table S2. Summary data of BIA analog SAR. Analog compound numbers with R_1/R_2 modifications on a constant 8-bromo core, chemical structures, NanoDSF thermal shifts (ΔT_i) reflecting binding to SLC1A1g, IC_{50} values calculated using the HEK293-SLC1A1 impedance assays, and GI_{50} values measured using Cell-Titer Glo. Data are presented as mean ± S.D. from the indicated number of biological replicates (n).

145 **Appendix Table S3. Structure activity relationship (b)**

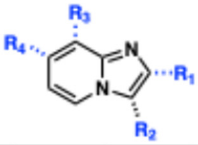

	R1	R2	R3	R4	NanoDSF ΔT_i (°C)	Impedance IC_{50} (µM)	384w CTG® GI_{50} (µM)
**3e**	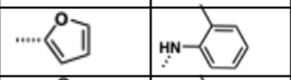		Br	H	+ 3.1 (n = 12)	9.7 (n = 9)	42 (n = 8)
**20**	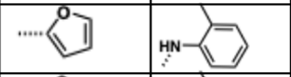		Br	F	+ 4.2 (n = 12)	4.1 (n = 4)	> 50 (n = 3)
**21**	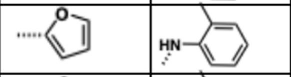		Br	CH_3	+ 2.9 (n = 6)	11 (n = 1)	32 (n = 3)
**22**	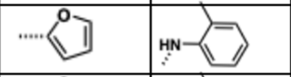		Br	OMe	+ 0.6 (n = 6)	n.d.	45 (n = 2)
**23**	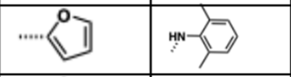		Br	F	+ 1.4 (n = 6)	48 (n = 1)	31 (n = 3)
**24**	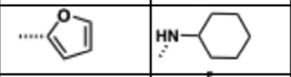		Br	F	+ 1.0 (n = 6)	n.d.	50 (n = 3)
**25**	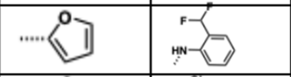		Br	F	+ 1.8 (n = 6)	19 (n = 1)	> 50 (n = 3)
**26**	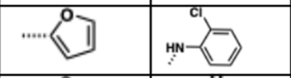		Br	F	+ 2.2 (n = 6)	25 (n =1)	> 50 (n = 3)
**27**	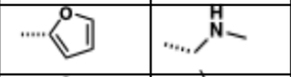		Br	F	- 0.3 (n = 6)	n.d.	28 (n = 6)
**28**	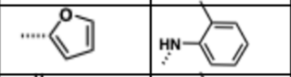		Me	F	+ 3.3 (n = 6)	6.9 (n = 2)	30 (n = 3)
**29**	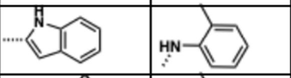		Br	F	+ 1.8 (n = 6)	10 (n = 1)	41 (n = 3)
**30 PBJ-001**	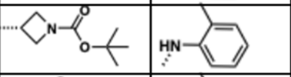		Br	F	+ 2.2 (n = 6)	9.2 (n = 2)	14 (n = 3)
**31**	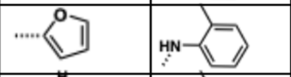		Me	H	+ 1.9 (n = 6)	14 (n =1)	30 (n = 3)
**32**	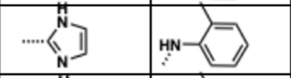		Br	F	+ 1.8 (n = 6)	28 (n = 2)	26 (n = 3)
**33**	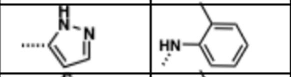		Br	F	+ 1.6 (n = 6)	39 (n = 1)	33 (n = 3)
**34**	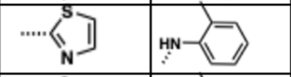		Br	F	+ 1.7 (n = 6)	21 (n =1)	42 (n = 3)
**35**	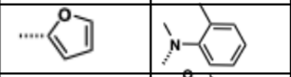		Br	F	+ 2.6 (n = 6)	8.8 (n = 1)	35 (n = 2)
**37**	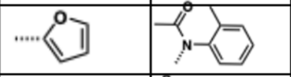		Br	F	- 0.3 (n = 6)	> 50 (n = 2)	> 50 (n = 2)
**37**	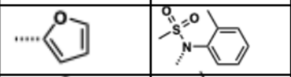		Br	F	- 0.1 (n = 6)	n.d.	> 50 (n = 2)
**38 PBJ-002**	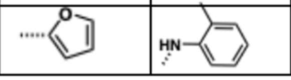		CHF_2	H	+ 2.6 (n = 6)	12 (n = 4)	22 (n = 2)

146

Appendix Table S3. Summary data of BIA analog SAR. Analog compound numbers and chemical structures of **3e** analogs with the indicated modifications in R_1—R_4, NanoDSF thermal shifts (*ΔT_i*) reflecting binding to SLC1A1g, IC_{50} values calculated using the HEK293-SLC1A1 impedance assays, and GI_{50} values measured using Cell-Titer Glo. Data are presented as mean ± S.D. from the indicated number of biological replicates (n).
