# Supplementary material for: Structure-guided optimization of SLC1A1/EAAT3-selective inhibitors targeting renal cancer metabolism
Source: EMBO J. 2026 Apr 22;45(11):3763–87. doi: 10.1038/s44318-026-00776-2 (PMC13226657; doi:10.1038/s44318-026-00776-2)
Supplement: Supplementary file 4 — Source data Fig. 3 [file 44318_2026_776_MOESM4_ESM.zip › Figure 3/3B/Multiple Alignment SLC1A Family.pdf]

- NCBI Home
- Sign in to NCBI
- Skip to Main Content
- Skip to Navigation
- About NCBI Accesskeys

NIH U.S. National Library of Medicine

NCBI National Center for Biotechnology Information

-
- My NCBI
- Sign in to NCBI
- Register
- Sign Out

COBALT Constraint-based Multiple Alignment Tool

- Home
- Recent Results
- Help

Phylogenetic Tree Edit and Resubmit Download

My Cobalt Results - Cobalt RID YZPMK4G0212 (5 seqs)

Graphical Overview

Find:

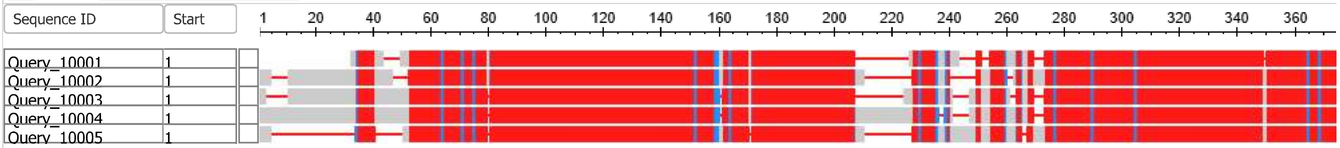

PROTEIN: 1 - 646 (646r shown)

Descriptions

Select All Re-align Alignment parameters

Accession	Description	Links
lcl|Query_10001	NP_004161 (EAAT3 SLC1A1) length=524	
lcl|Query_10002	ENST00000278379.9 (EAAT2 SLC1A2) length=574	
lcl|Query_10003	ENST00000265113.9 (EAAT1 SLC1A3) length=542	
lcl|Query_10004	ENST00000594383.2 (EAAT4 SLC1A6) length=564	
lcl|Query_10005	ENST00000371494.9 (EAAT5 SLC1A7) length=560	

Alignments

Select All Re-align Mouse over the sequence identifer for sequence title

View Format: Compact Conservation Setting: 2 Bits

```
Query_10001  1    ----   ----------------------MGKPARKGCEW------KRFLKNNWVLLSTVAAVVLGITTGVLVREHsNLS  45
Query_10002  1    MAST   EGANNMPKQVEVRMHDSHLGSEEPKHRHLGLRLCDK------LGKNLLLTLTVFGVILGAVCGGLLRLAsPIH  71
Query_10003  1    MT--   KSNGEEPKMGGRMERFQQGVRKRTLLAKKKVQNITKedvKSYLFRNAFVLLTVTAVIVGTILGFTLRPY-RMS  74
Query_10004  1    MSSH[6]RESGQRLGRVGWLQRLQESLQQRALRTRLRLQTMTLehvLRFLRRNAFILLTVSAVVIGVSLAFALRPY-QLT  82
Query_10005  1    MVPH   ------------------------AILARGR----------DVCRRNGLLILSVLSVIVGCLLGFFLRTR-RLS  43

Query_10001  46   TLEKFYFAFPGEILMRMLKLIILPLIISSMITGVAALDSNVSGKIGLRAVVYYFCTTLIAVILGIVLVVSIKPGVTQKVG  125
Query_10002  72   PDVVMLIAFPGDILMRMLKMLILPLIISSLITGLSGLDAKASGRLGTRAMVYYMSTTIIAAVLGVILVLAIHPGNPKLKK  151
Query_10003  75   YREVKYFSFPGELLMRMLQMLVLPLIISSLVTGMAALDSKASGKMGMRAVVYYMTTTIIAVVIGIIIVIIIHPGKGT-KE  153
```

```
Query_10004  83   YRQIKYFSFPGELLMRMLQMLVLPLIVSSLVTGMASLDNKATGRMGMRAAVYYMVTTIIAVFIGILMVTIIHPGKGS-KE  161
Query_10005  44   PQEISYFQFPGELLMRMLKMMILPLVVSSLMSGLASLDAKTSSRLGVLTVAYYLWTTFMAVIVGIFMVSIIHPGSAAQKE  123

Query_10001  126  EIARTGSTPEVSTVDAMLDLIRNMFPENLVQACFQQYKTKREEV---    KPPSDPEMNMTEESF[2]--VM---TTAI  192
Query_10002  152  QLGPGKKNDEVSSLDAFLDLIRNLFPENLVQACFQQIQTVTKKVLVA    -PPPDEEANATSAV-   --VSLLNETVT  220
Query_10003  154  NMHREGKIVRVTAADAFLDLIRNMFPPNLVEACFKQFKTNYEKR---[ 2]KVPIQANETLVGAVI   NNVSEAMETLT  225
Query_10004  162  GLHREGRIETIPTADAFMDLIRNMFPPNLVEACFKQFKTQYSTRVVT[16]PPPFSVENGT--SFL   ENVTRALGTLQ  248
Query_10005  124  TTEQSGK-PIMSSADALLDLIRNMFPANLVEATFKQYRTKTTPVVKS    -PKVAPEEAPPRRIL[6]ENGSHVQNFAL  200

Query_10001  193  SKNKTKEYKIV   GMYSDGINVLGLIVFCLVFGLVIGKMGEKGQILVDFFNALSDATMKIVQIIMCYMPLGILFLIAGK  269
Query_10002  221  EV--PEETKMV[4]LEFKDGMNVLGLIGFFIAFGIAMGKMGDQAKLMVDFFNILNEIVMKLVIMIMWYSPLGIACLICGK  299
Query_10003  226  RIT--EELVPV   PGSVNGVNALGLVVFSMCFGFVIGNMKEQGQALREFFDSLNEAIMRLVAVIMWYAPVGILFLIAGK  300
Query_10004  249  EMLSFEETVPV   PGSANGINALGLVVFSVAFGLVIGGMKHKGRVLRDFFDSLNEAIMRLVGIIIWYAPVGILFLIAGK  325
Query_10005  201  DLTPPPE--VV[4]PGTSDGMNVLGIVFFSATMGIMLGRMGDSGAPLVSFCQCLNESVMKIVAVAVWYFPFGIVFLIAGK  279

Query_10001  270  IIEVEDWEIF-RKLGLYMATVLTGLAIHSIVILPLIYFIVVRKNPFRFAMGMAQALLTALMISSSSATLPVTFRCAEENN  348
Query_10002  300  IIAIKDLEVVARQLGMYMVTVIIGLIIHGGIFLPLIYFVVTRKNPFSFFAGIFQAWITALGTASSAGTLPVTFRCLEENL  379
Query_10003  301  IVEMEDMGVIGGQLAMYTVTVIVGLLIHAVIVLPLLYFLVTRKNPWVFIGGLLQALITALGTSSSSATLPITFKCLEENN  380
Query_10004  326  ILEMEDMAVLGGQLGMYTLTVIVGLFLHAGIVLPLIYFLVTHRNPFPFIGGMLQALITAMGTSSSSATLPITFRCLEEGL  405
Query_10005  280  ILEMDDPRAVGKKLGFYSVTVVCGLVLHGLFILPLLYFFITKKNPIVFIRGILQALLIALATSSSSATLPITFKCLLENN  359

Query_10001  349  QVDKRITRFVLPVGATINMDGTALYEAVAAVFIAQLNDLDLGIGQIITISITATSASIGAAGVPQAGLVTMVIVLSAVGL  428
Query_10002  380  GIDKRVTRFVLPVGATINMDGTALYEAVAAIFIAQMNGVVLDGGQIVTVSLTATLASVGAASIPSAGLVTMLLILTAVGL  459
Query_10003  381  GVDKRVTRFVLPVGATINMDGTALYEALAAIFIAQVNNFELNFGQIITISITATAASIGAAGIPQAGLVTMVIVLTSVGL  460
Query_10004  406  GVDRRITRFVLPVGATVNMDGTALYEALAAIFIAQVNNYELNLGQITTISITATAASVGAAGIPQAGLVTMVIVLTSVGL  485
Query_10005  360  HIDRRIARFVLPVGATINMDGTALYEAVAAIFIAQVNNYELDFGQIITISITATAASIGAAGIPQAGLVTMVIVLTSVGL  439

Query_10001  429  PAEDVTLIIAVDWLLDRFRTMVNVLGDAFGTGIVEKLSKKELEQMDVSSEVNivNPFALESTI--LDNEDS-DTKKSYVN  505
Query_10002  460  PTEDISLLVAVDWLLDRMRTSVNVVGDSFGAGIVYHLSKSELDTIDSQHRVH--EDIEMTKTQS--IYDDMkNHRESNSN  535
Query_10003  461  PTDDITLIIAVDWFLDRLRTTTNVLGDSLGAGIVEHLSRHELKNRDVEMGNSviEENEMKKPYQ-LIAQDN-ETEKPIDS  538
Query_10004  486  PTEDITLIIAVDWFLDRLRTMTNVLGDSIGAAVIEHLSQRELELQEAELTLP-----SLGKPYKsLMAQEKgASRGRGGN  560
Query_10005  440  PTDDITLIIAVDWALDRFRTMINVLGDALAAGIMAHICRKDF------------------------ARDT-GTEKLLPC  493

Query_10001  506  GGFAVDKSDTISFTQTSQF      524
Query_10002  536  QCVYAAHNSVIVDECKVTL[20]  574
Query_10003  539  ETKM---------------      542
Query_10004  561  ESAM---------------      564
Query_10005  494  ETKPVSLQEIVAAQQNGCV[48]  560
```

BLAST is a registered trademark of the National Library of Medicine.
Copyright | Disclaimer | Privacy | Accessibility | Contact | Send feedback

NCBI | NLM | NIH | DHHS

HHS Vulnerability Disclosure
