# Supplementary figures and images for: Structure-guided optimization of SLC1A1/EAAT3-selective inhibitors targeting renal cancer metabolism
Source: EMBO J. 2026 Apr 22;45(11):3763–87. doi: 10.1038/s44318-026-00776-2 (PMC13226657; doi:10.1038/s44318-026-00776-2)

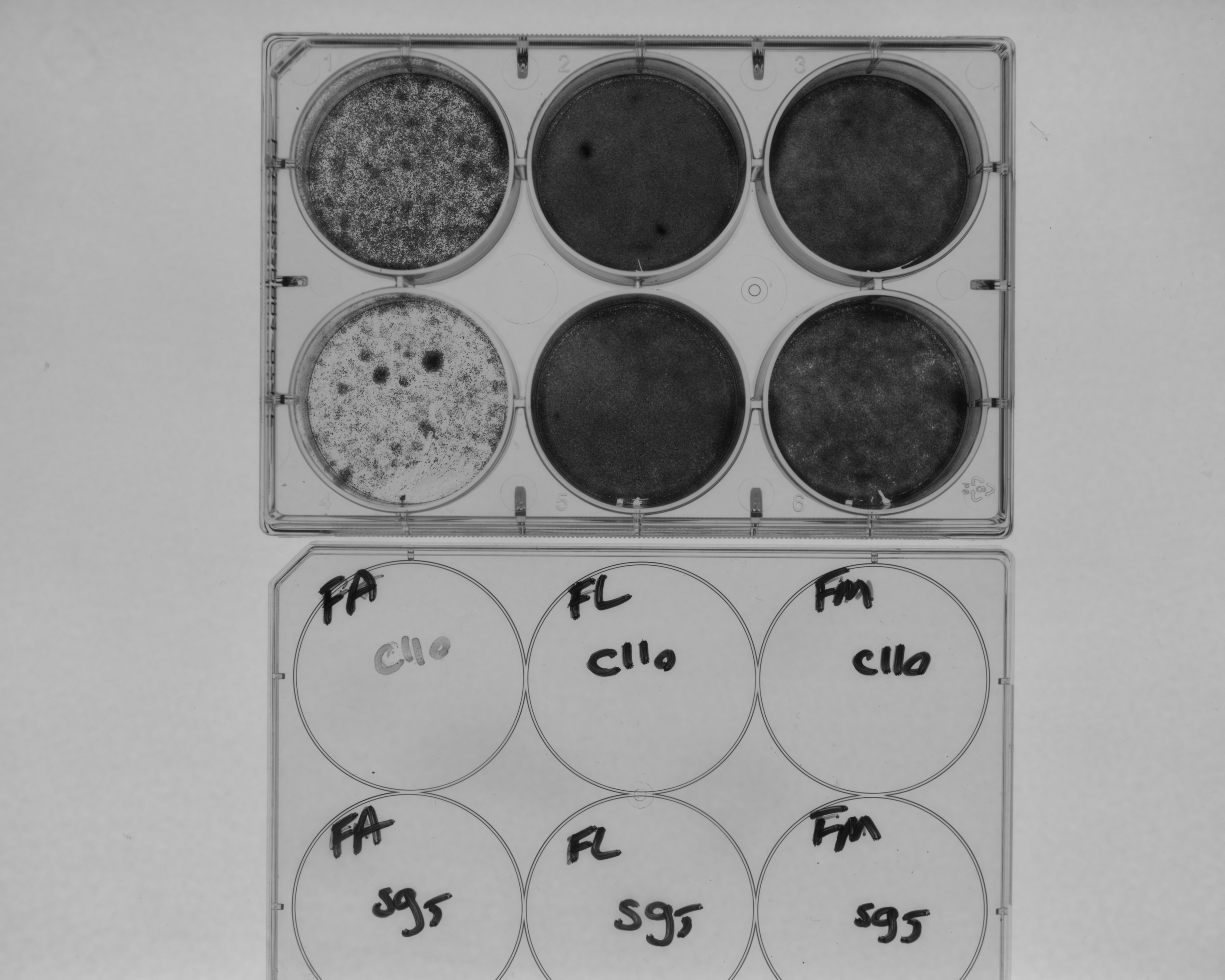

Supplement: Supplementary file 4 — Source data Fig. 3 [file 44318_2026_776_MOESM4_ESM.zip › Figure 3/3C/Chakraborty lab 2024-09-16 12h56m47s(Coomassie Blue).raw16.tif]

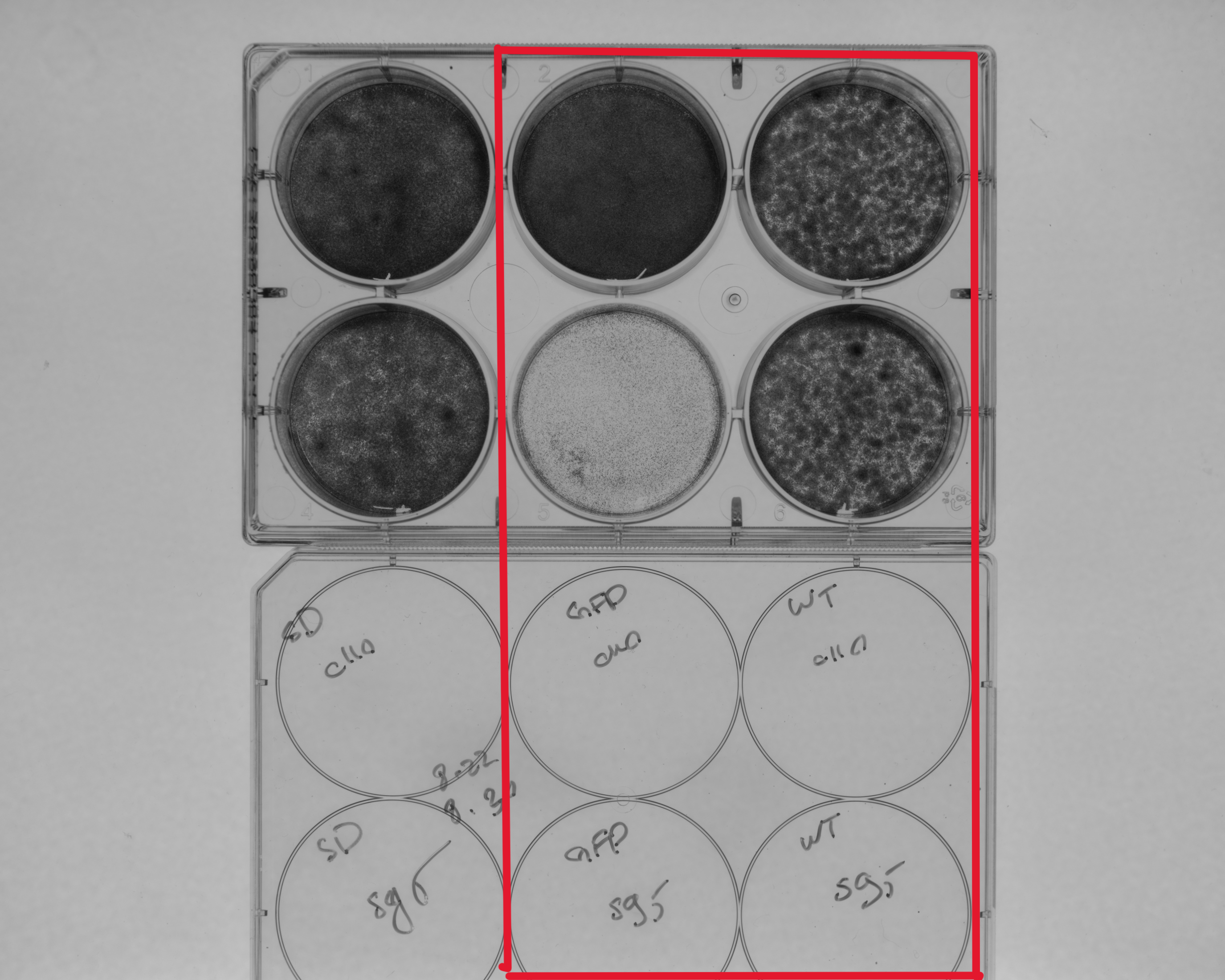

Supplement: Supplementary file 4 — Source data Fig. 3 [file 44318_2026_776_MOESM4_ESM.zip › Figure 3/3C/Chakraborty lab 2024-09-16 12h57m56s(Coomassie Blue).raw16.tif]

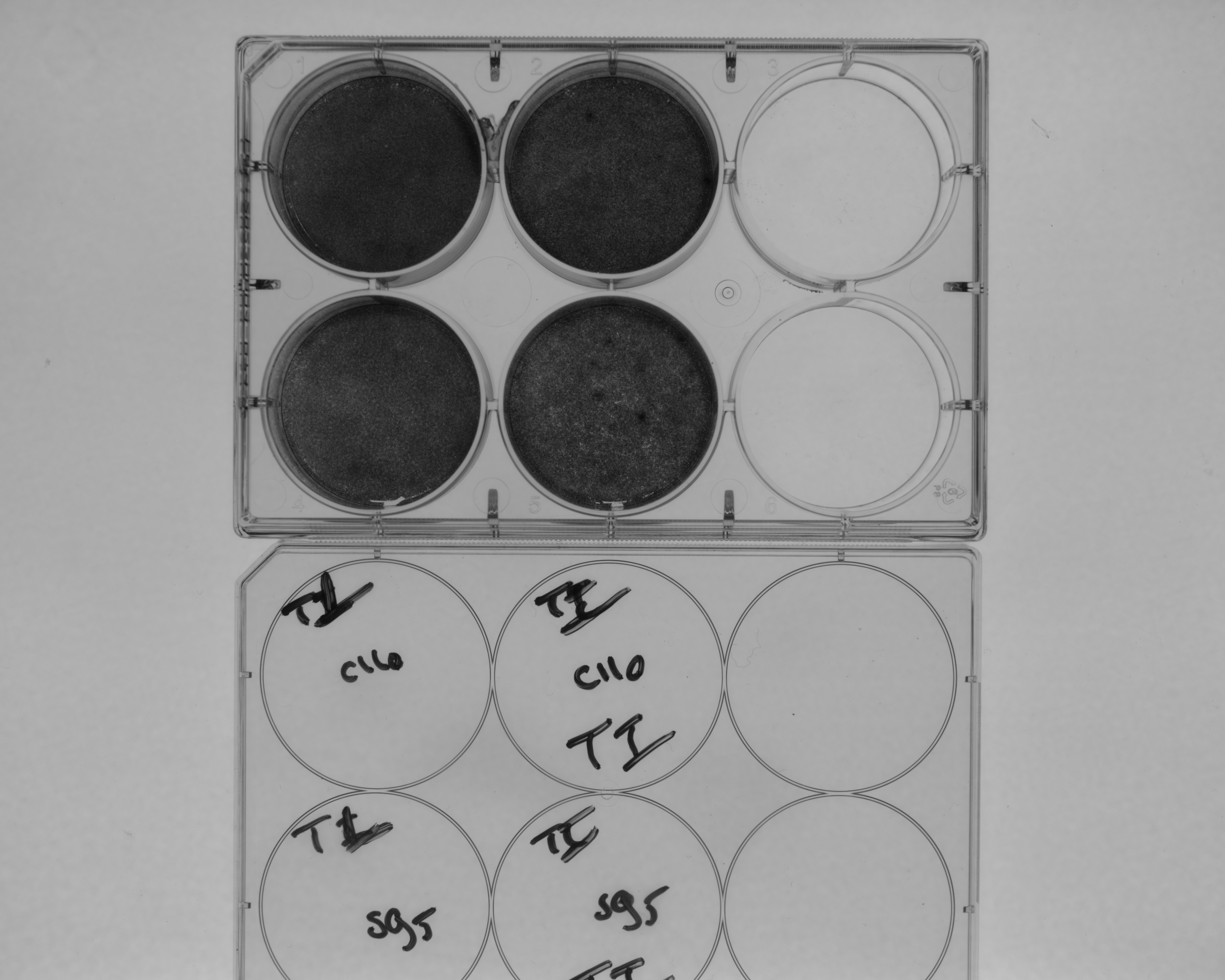

Supplement: Supplementary file 4 — Source data Fig. 3 [file 44318_2026_776_MOESM4_ESM.zip › Figure 3/3C/Chakraborty lab 2024-09-16 12h58m58s(Coomassie Blue).raw16.tif]

## Slide 1
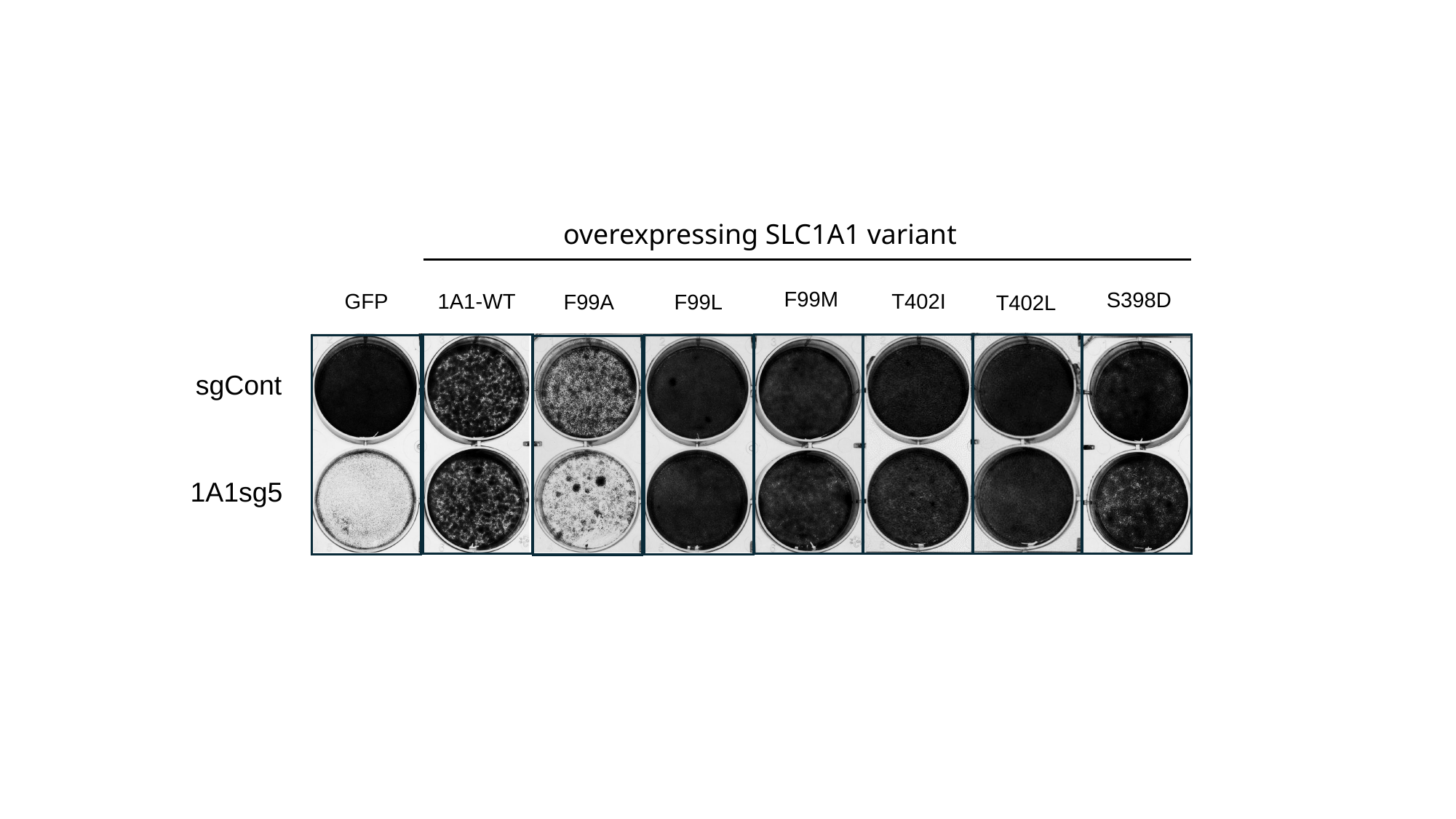

overexpressing SLC1A1 variant
F99M
S398D
GFP
1A1-WT
T402I
F99L
F99A
T402L
sgCont
1A1sg5

Supplement: Supplementary file 4 — Source data Fig. 3 [file 44318_2026_776_MOESM4_ESM.zip › Figure 3/3C/combining 3 pictures.pptx]

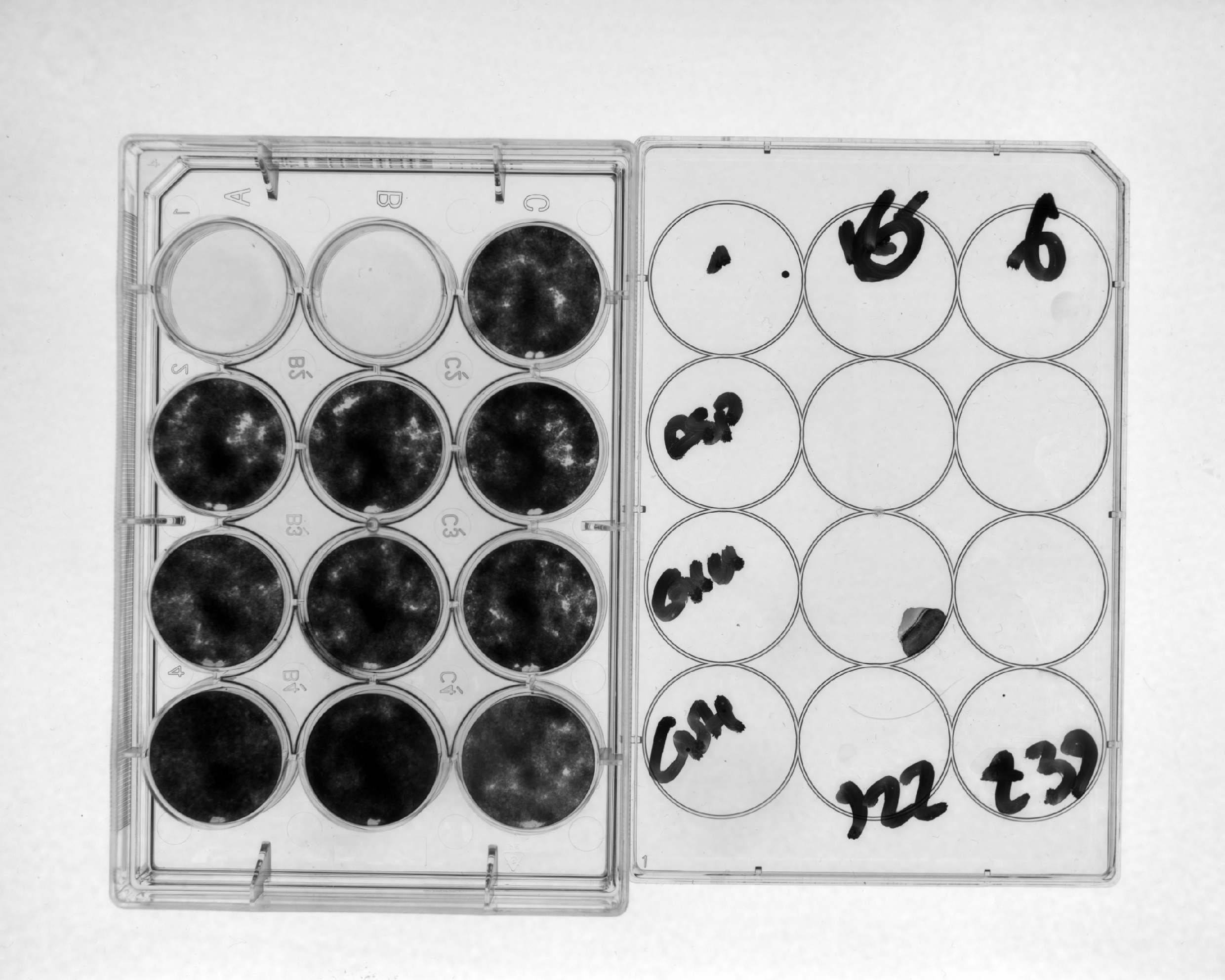

Supplement: Supplementary file 8 — Source data Fig. 7 [file 44318_2026_776_MOESM8_ESM.zip › Figure 7/7I/Chakraborty lab 2025-05-06 16h43m10s(Coomassie Blue).tif]

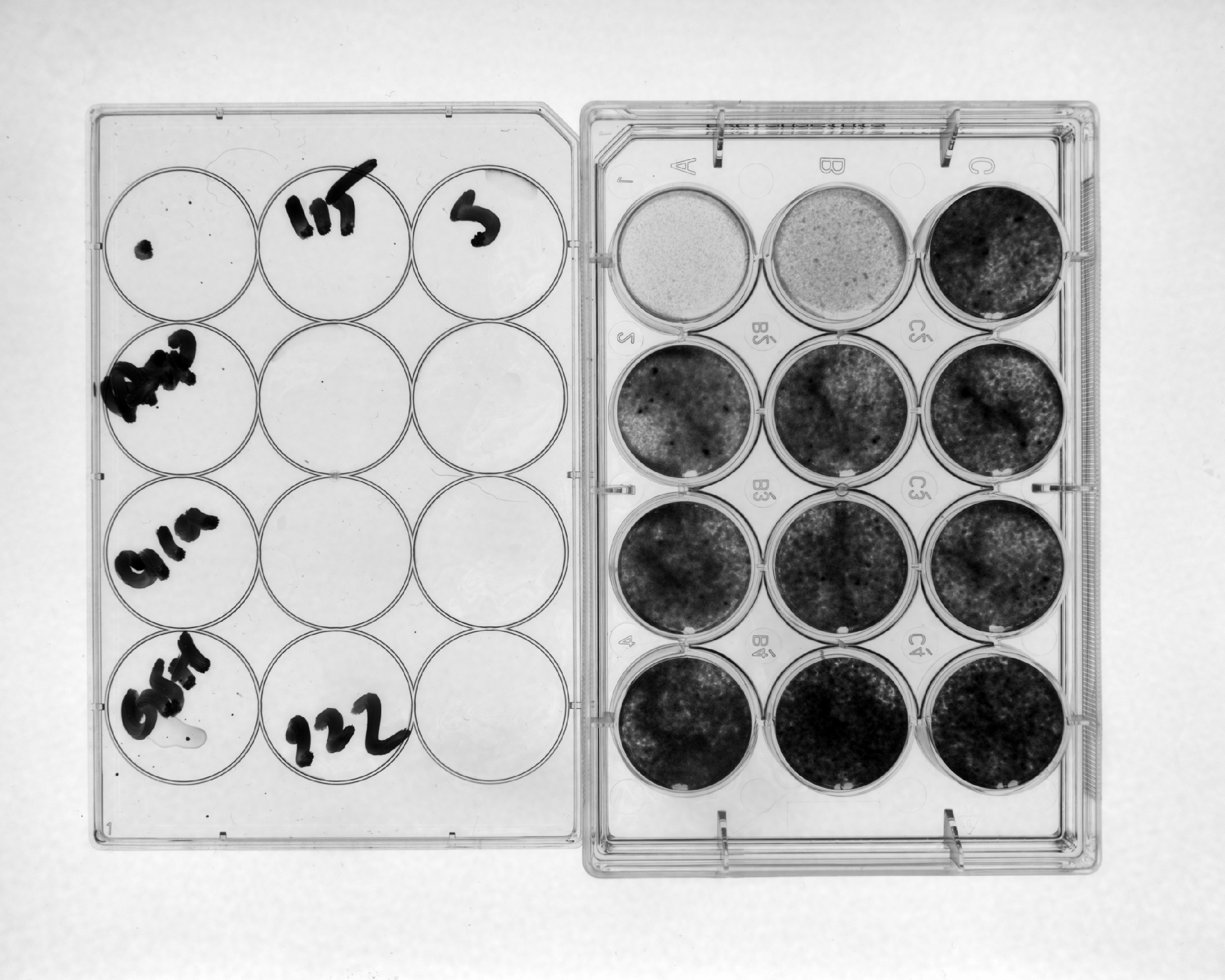

Supplement: Supplementary file 8 — Source data Fig. 7 [file 44318_2026_776_MOESM8_ESM.zip › Figure 7/7I/Chakraborty lab 2025-05-06 16h44m17s(Coomassie Blue).tif]

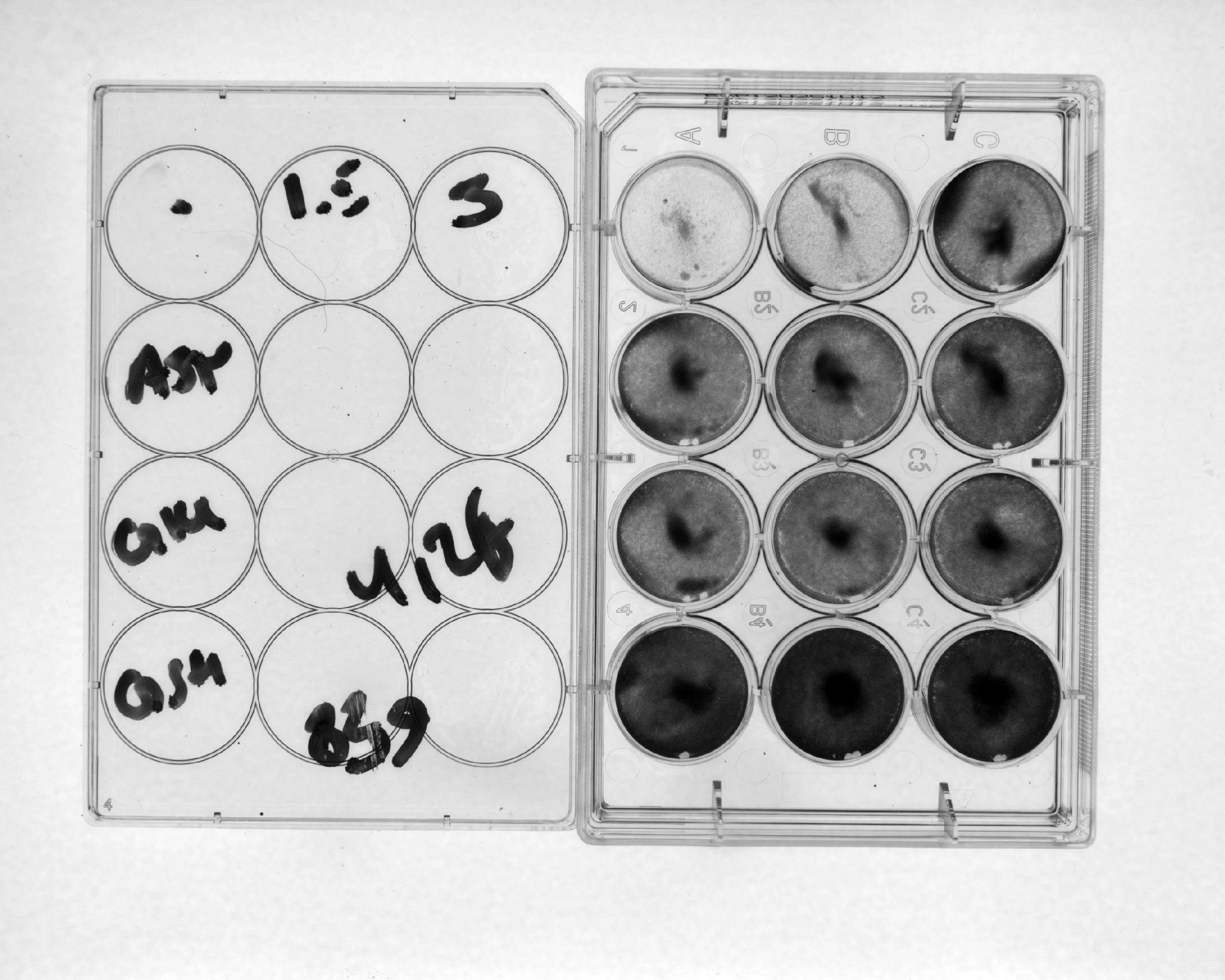

Supplement: Supplementary file 8 — Source data Fig. 7 [file 44318_2026_776_MOESM8_ESM.zip › Figure 7/7I/Chakraborty lab 2025-05-06 16h45m23s(Coomassie Blue).tif]
