# Supplementary material for: Structure-guided optimization of SLC1A1/EAAT3-selective inhibitors targeting renal cancer metabolism
Source: EMBO J. 2026 Apr 22;45(11):3763–87. doi: 10.1038/s44318-026-00776-2 (PMC13226657; doi:10.1038/s44318-026-00776-2)
Supplement: Supplementary file 6 — Source data Fig. 5 [file 44318_2026_776_MOESM6_ESM.zip › Figure 5/5I-K/synergyfinder+_report.pdf]

SynergyFinder+ Report

https://synergyfinder.org/ http://synergyfinder.ai/ www.synergyfinderplus.org
https://tangsoftwarelab.shinyapps.io/synergyfinder

2025-12-10

```
## Correct Baseline Setting:  non
```

Data tables

Table 1: Drug Combiantion Meta Data

Block ID	$Drug_1$	$Drug_2$	Conc $Unit_1$	Conc $Unit_2$
1	3e	PT2385	uM	uM
2	PBJ1	PT2385	uM	uM
3	PBJ2	PT2385	uM	uM

Table 2: Synergy Score Summary Table

Block ID	ZIP	Loewe	HSA	Bliss
1	2.62	10.83	12.87	3.87
2	21.77	27.56	28.82	20.45
3	16.11	22.58	22.72	16.09

Table 3: Synergy Score Statistic Table

Block ID	ZIP p Value	Loewe p Value	HSA p Value	Bliss p Value
1	5.20e-01	1.34e-03	4.12e-05	3.34e-01
2	2.93e-06	8.16e-18	1.55e-12	5.30e-04
3	3.96e-11	2.43e-43	4.17e-51	3.91e-14

Reference

For use of the SynergyFinder+ R package or the web application:

[1] Zheng, S.; Wang, W.; Aldahdooh, J.; Malyutina, A.; Shadbahr, T.; Tanoli, Z.; Passia, A.; Tang, J. SynergyFinder Plus: Toward Better Interpretation and Annotation of Drug Combination Screening Datasets. Genomics, Proteomics & Bioinformatics 2022, 20 (3), 587-596. doi:10.1016/j.gpb.2022.01.004.

For use of ZIP synergy scoring:

[2] Yadav, B.; Wennerberg, K.; Aittokallio, T.; Tang, J. Searching for Drug Synergy in Complex Dose-Response Landscapes Using an Interaction Potency Model. Comput Struct Biotechnol J 2015, 13, 504–513. doi:10.1016/j.csbj.2015.09.001

For how to harmonize the different synergy scoring methods:

[3] Tang, J.; Wennerberg, K.; Aittokallio, T. What Is Synergy? The Saariselkä Agreement Revisited. Front Pharmacol 2015, 6, 181. doi:10.3389/fphar.2015.00181

For general ideas of drug combination therapies:

[4] Tang, J. Informatics Approaches for Predicting, Understanding, and Testing Cancer Drug Combinations. Methods Mol Biol 2017, 1636, 485–506. doi:10.1007/978-1-4939-7154-1_30

For retrieving the most comprehensive drug combination data resources and their sensitivity and synergy results by SynergyFinder, please go to DrugComb :

[5] Zheng, S.; Aldahdooh, J.; Shadbahr, T.; Wang, Y.; Aldahdooh, D.; Bao, J.; Wang, W.; Jing, T. DrugComb update: a more comprehensive drug sensitivity data repository and analysis portal. Nucleic Acids Research 2021, 49 (w1), w174-w184. doi:10.1093/nar/gkab438

[6] Zagidullin, B.; Aldahdooh, J.; Zheng, S.; Wang, W.; Wang, Y.; Saad, J.; Malyutina, A.; Jafari, M.; Tanoli, Z.; Pessia, A.; Tang, J. DrugComb: An Integrative Cancer Drug Combination Data Portal. Nucleic Acids Res 2019, 47 (W1), W43–W51. doi:10.1093/nar/gkz337

For use of combination sensitivity score:

[7] Malyutina, A.; Majumder, M. M.; Wang, W.; Pessia, A.; Heckman, C. A.; Tang, J. Drug Combination Sensitivity Scoring Facilitates the Discovery of Synergistic and Efficacious Drug Combinations in Cancer. PLOS Computational Biology 2019, 15 (5), e1006752. doi:10.1371/journal.pcbi.1006752

Dose-Response Curve

3e in Block 1

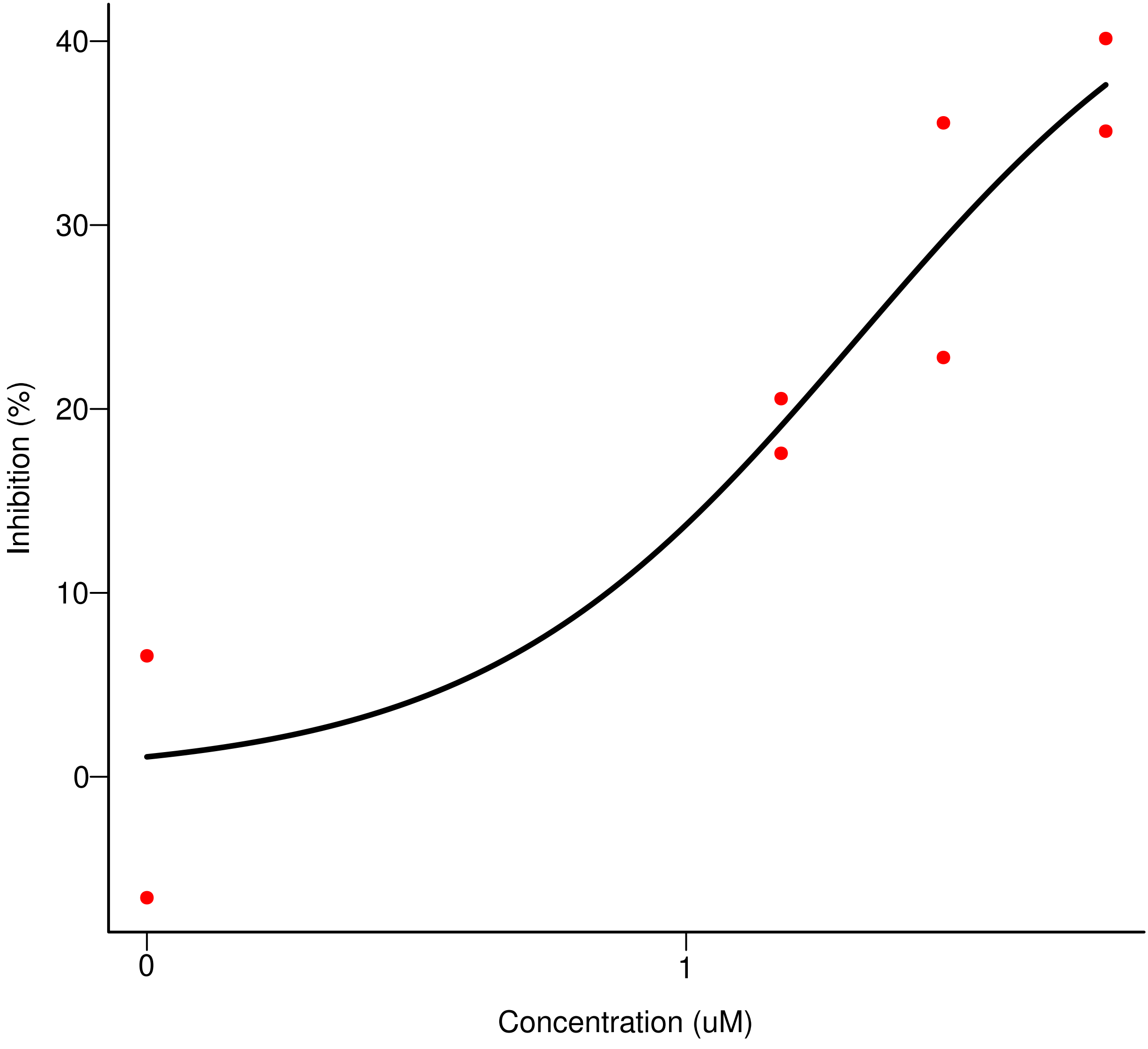

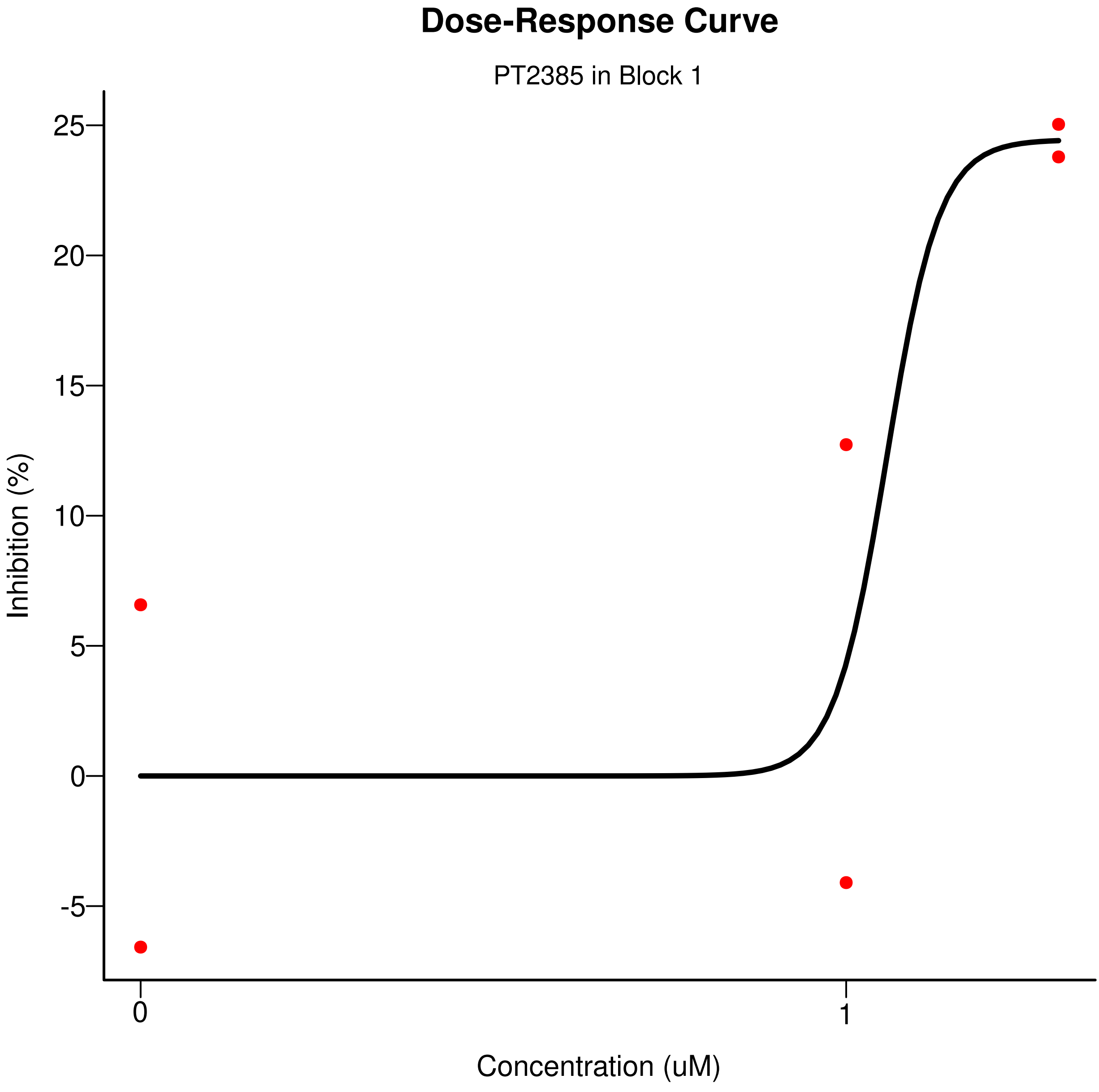

Dose-Response Curve
PT2385 in Block 1
Inhibition (%)
25
20
15
10
5
0
-5
0
1
Concentration (uM)

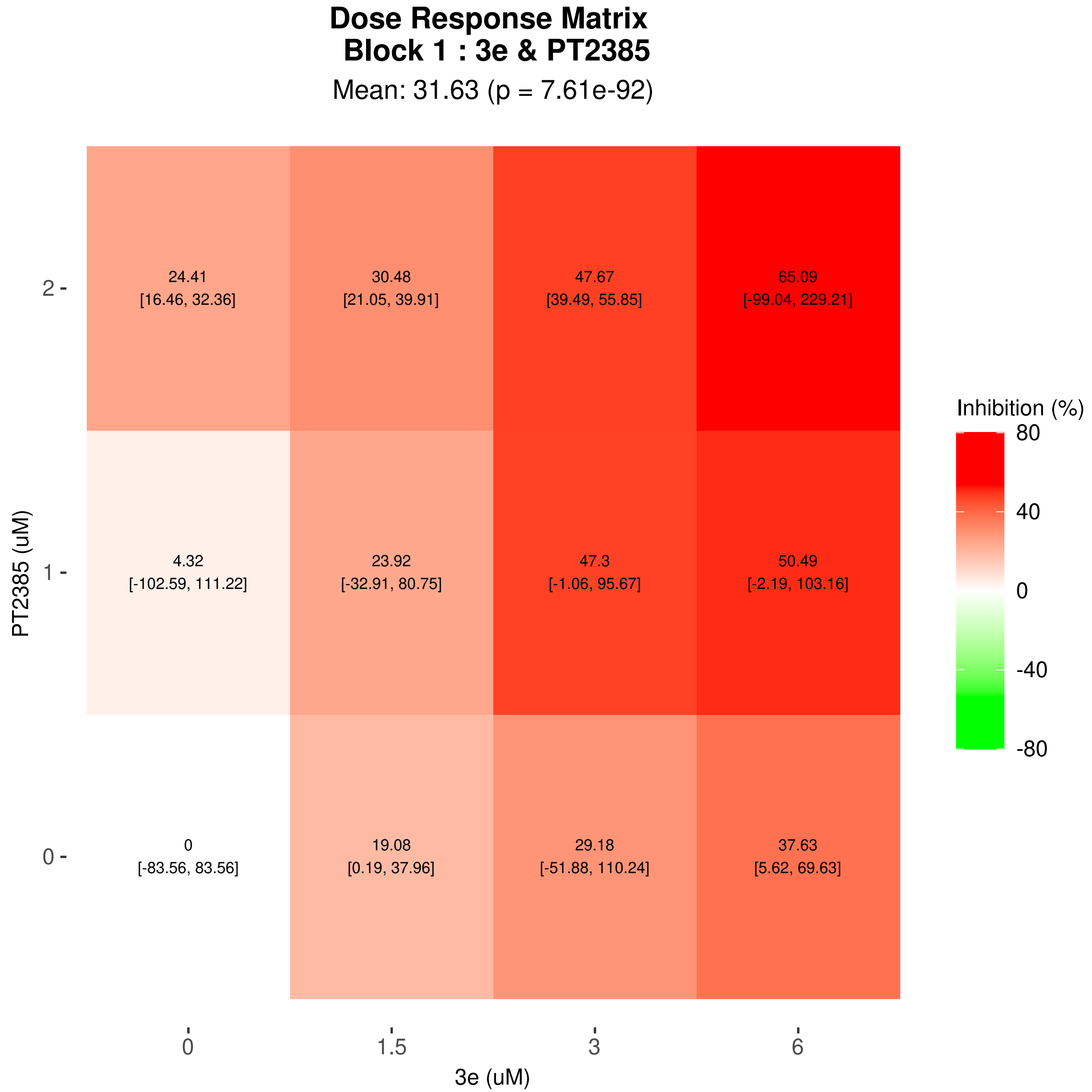
Dose Response Matrix
Block 1 : 3e & PT2385
Mean: 31.63 (p = 7.61e-92)
24.41
[16.46, 32.36]
30.48
[21.05, 39.91]
47.67
[39.49, 55.85]
65.09
[-99.04, 229.21]
4.32
[-102.59, 111.22]
23.92
[-32.91, 80.75]
47.3
[-1.06, 95.67]
50.49
[-2.19, 103.16]
0
[-83.56, 83.56]
19.08
[0.19, 37.96]
29.18
[-51.88, 110.24]
37.63
[5.62, 69.63]
PT2385 (uM)
2
1
0
0
1.5
3
6
3e (uM)
Inhibition (%)
80
40
0
-40
-80

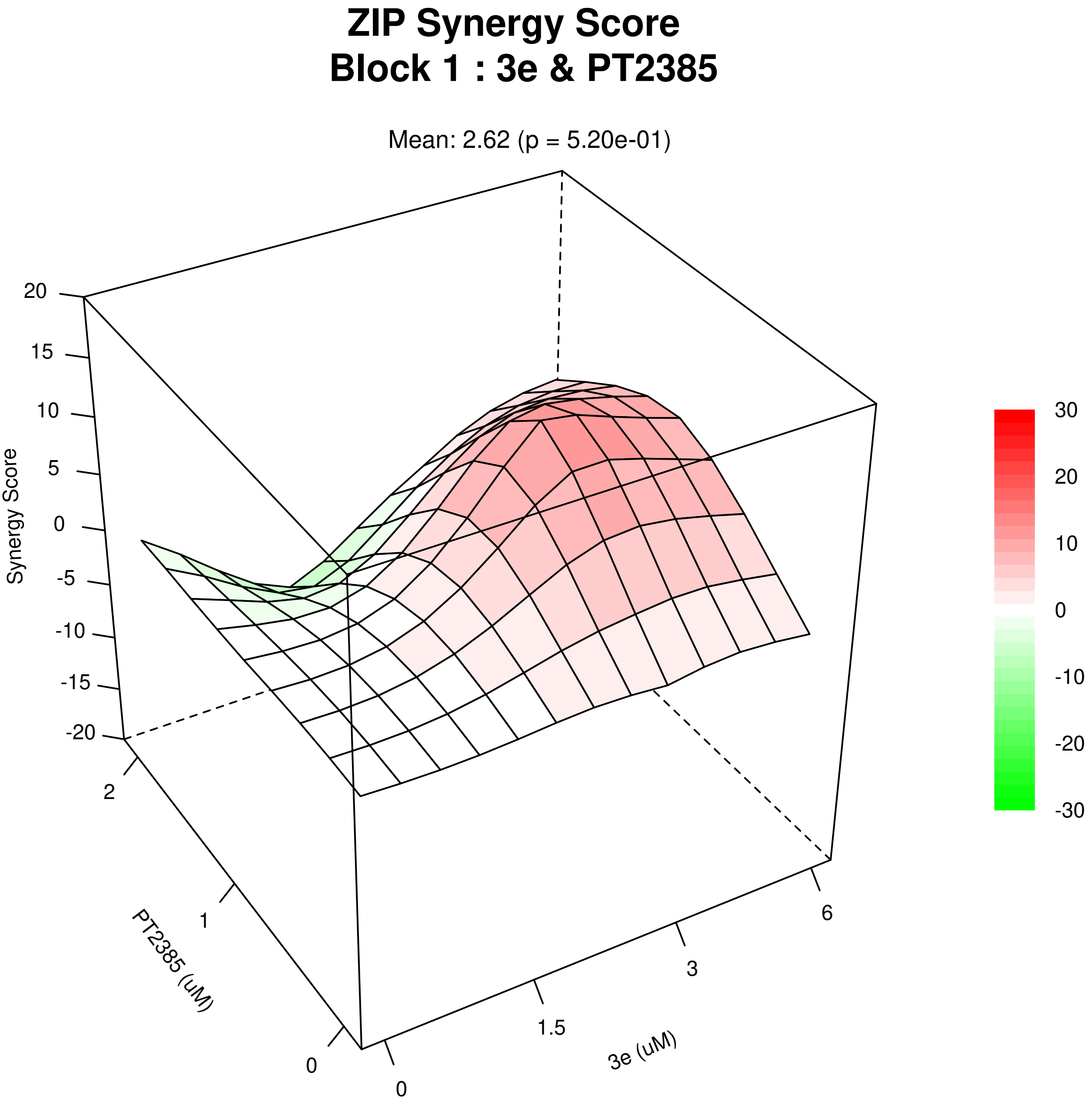
ZIP Synergy Score
Block 1 : 3e & PT2385
Mean: 2.62 (p = 5.20e-01)
Synergy Score
20
15
10
5
0
-5
-10
-15
-20
PT2385 (uM)
2
1
0
3e (uM)
0
1.5
3
6
30
20
10
0
-10
-20
-30

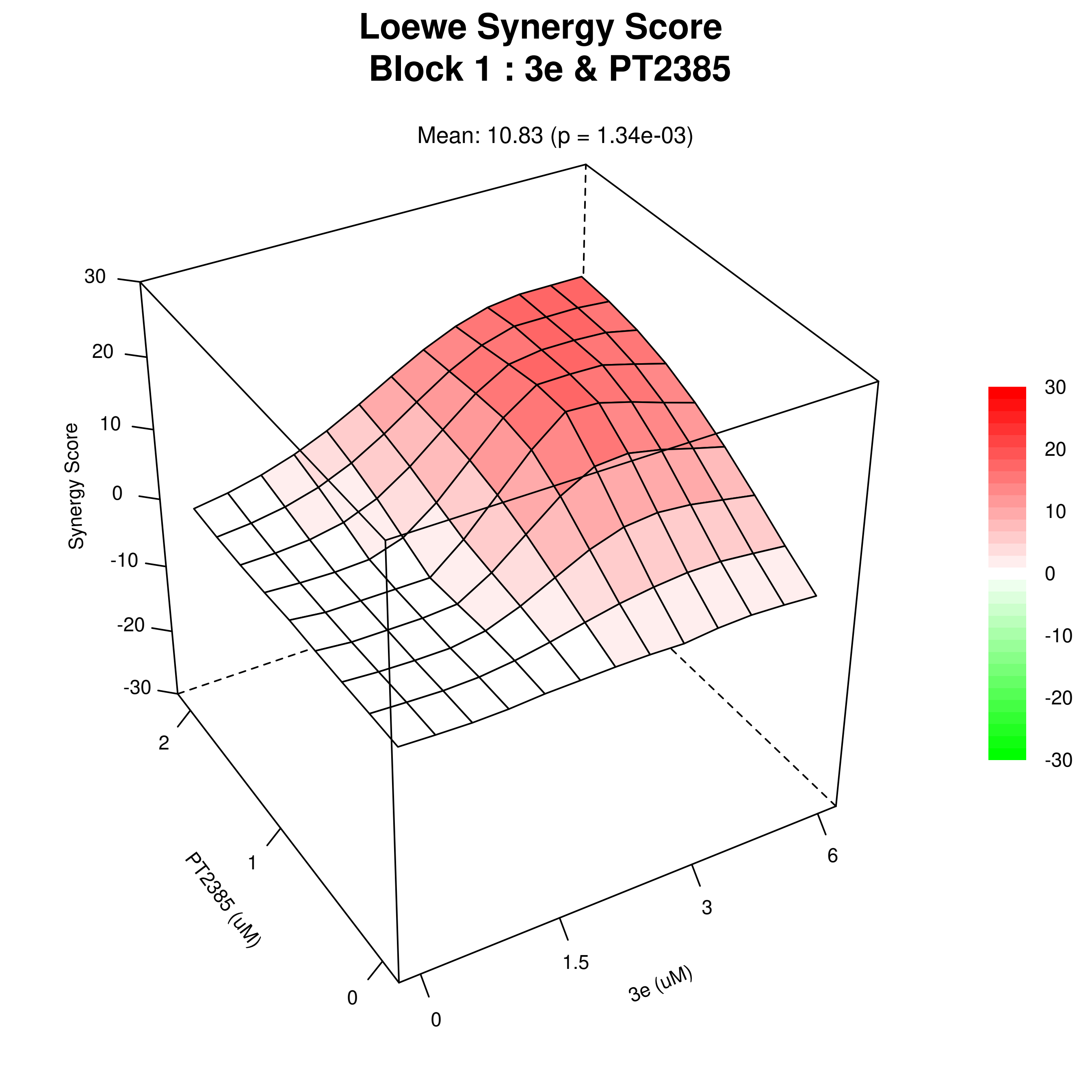

Loewe Synergy Score
Block 1 : 3e & PT2385
Mean: 10.83 (p = 1.34e-03)
Synergy Score
30
20
10
0
-10
-20
-30
PT2385 (uM)
2
1
0
3e (uM)
0
1.5
3
6
30
20
10
0
-10
-20
-30

Bliss Synergy Score
Block 1 : 3e & PT2385

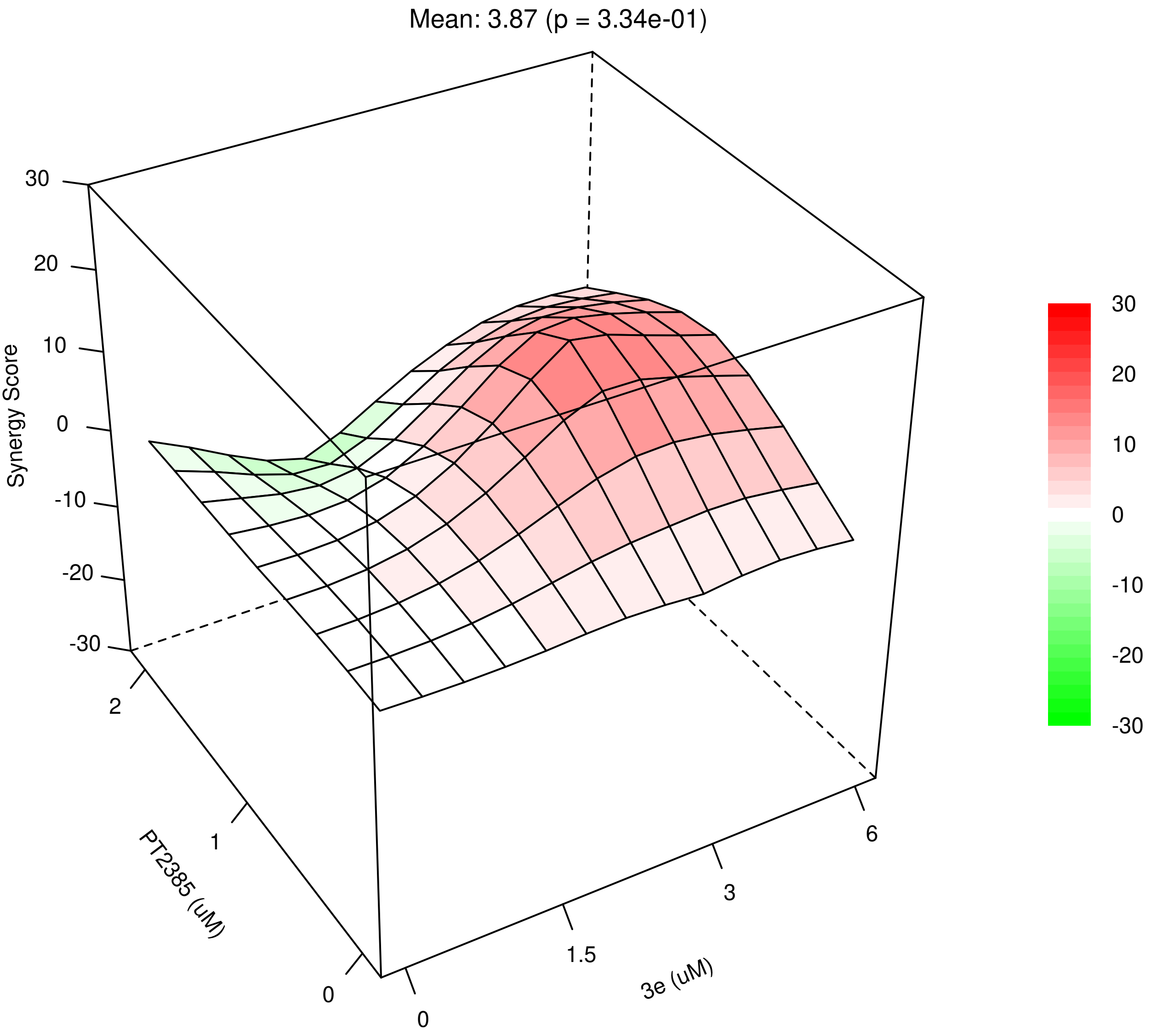

HSA Synergy Score
Block 1 : 3e & PT2385

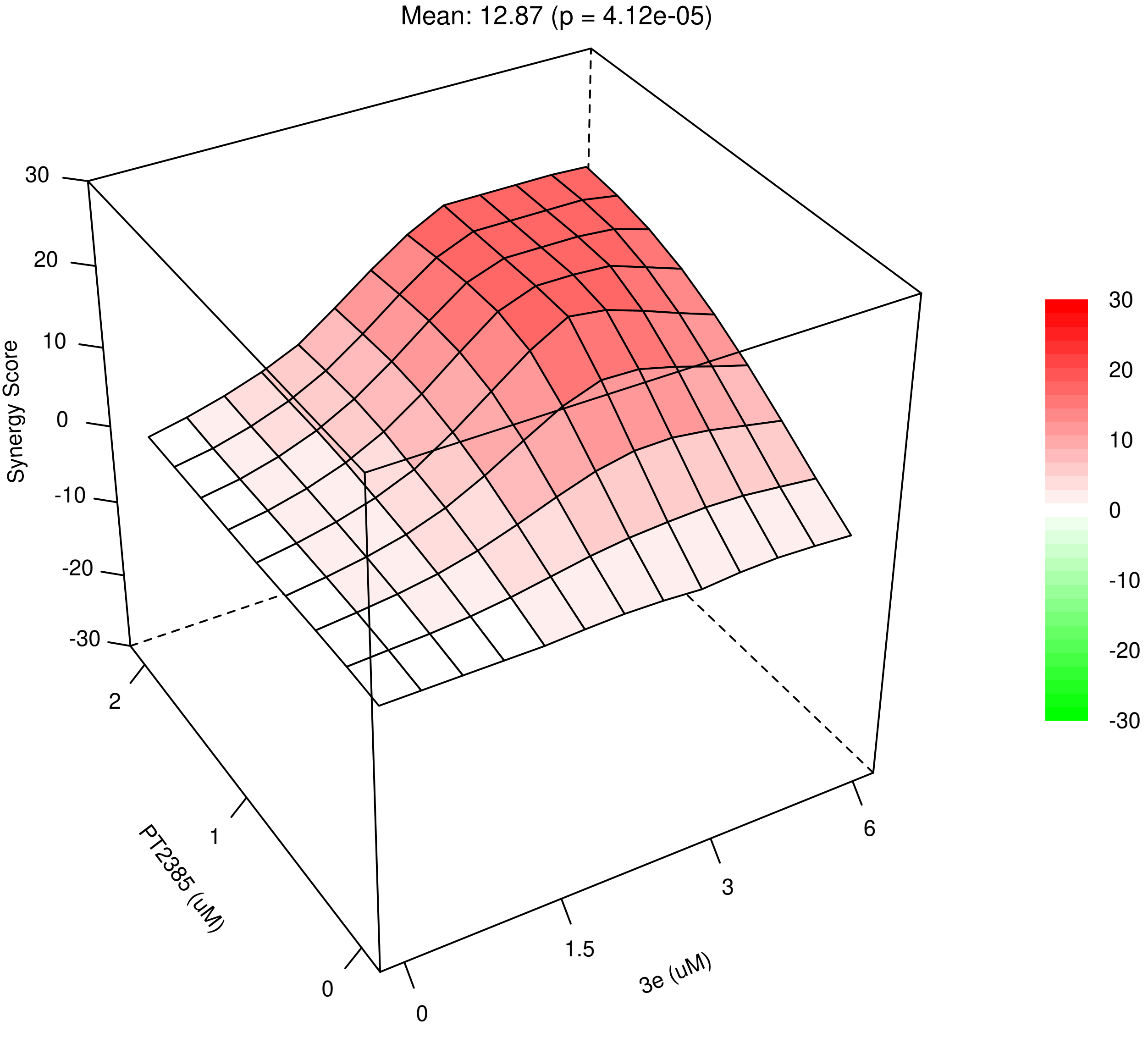

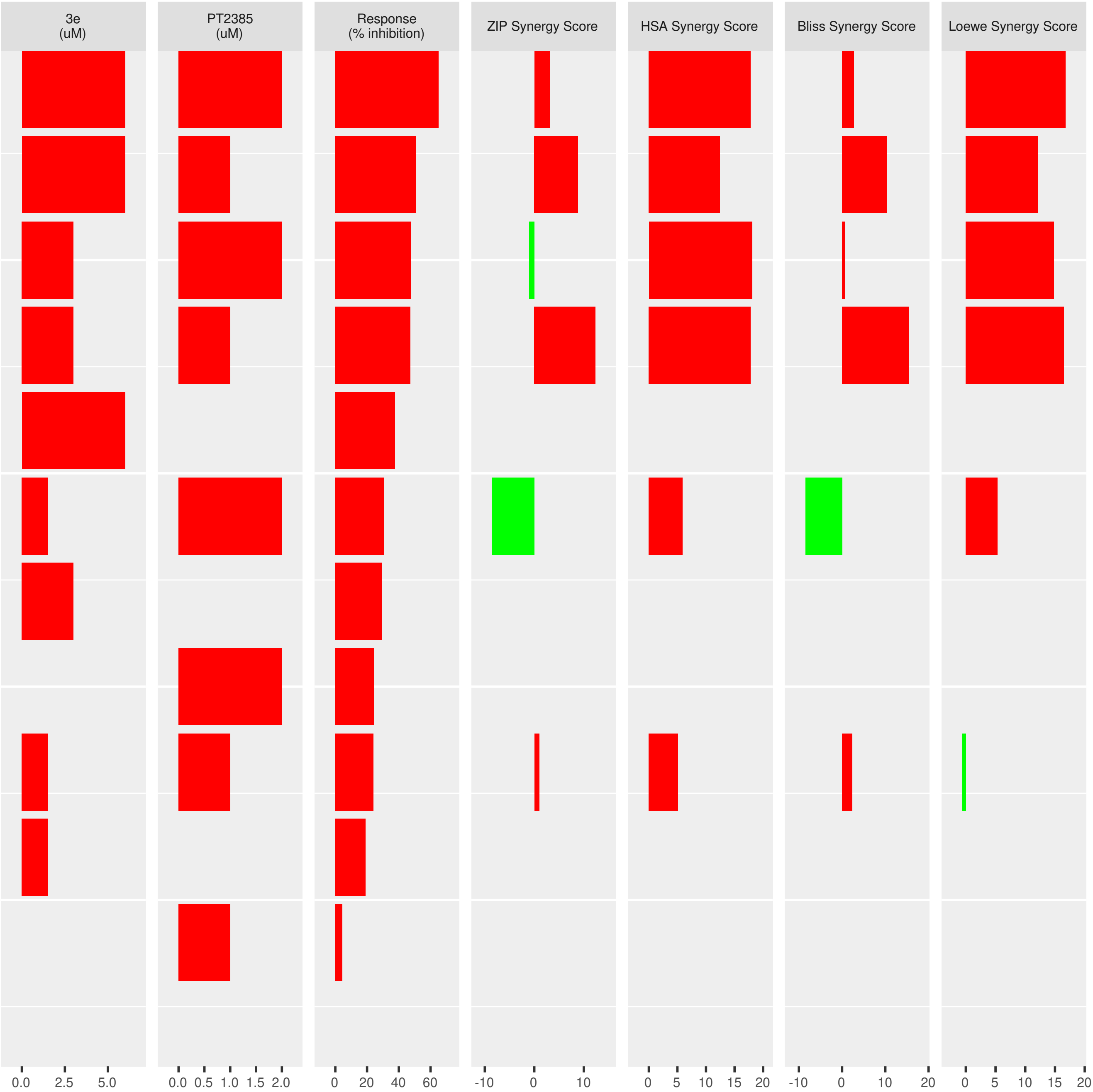
3e
(uM)
PT2385
(uM)
Response
(% inhibition)
ZIP Synergy Score
HSA Synergy Score
Bliss Synergy Score
Loewe Synergy Score
0.0
2.5
5.0
0.0
0.5
1.0
1.5
2.0
0
20
40
60
-10
0
10
0
5
10
15
20
-10
0
10
20
0
5
10
15
20

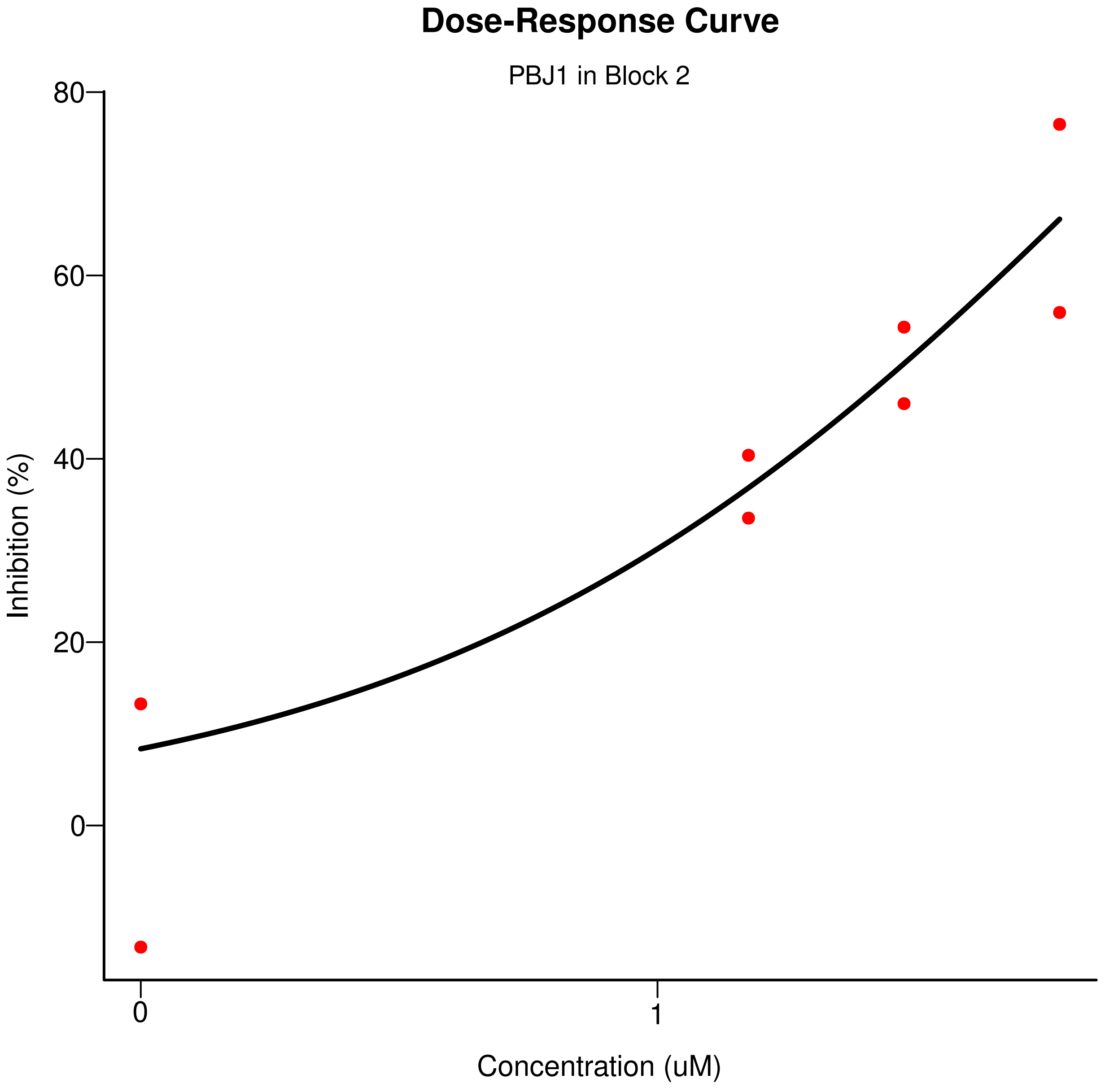

Dose-Response Curve
PBJ1 in Block 2
80
60
40
20
0
Inhibition (%)
0
1
Concentration (uM)

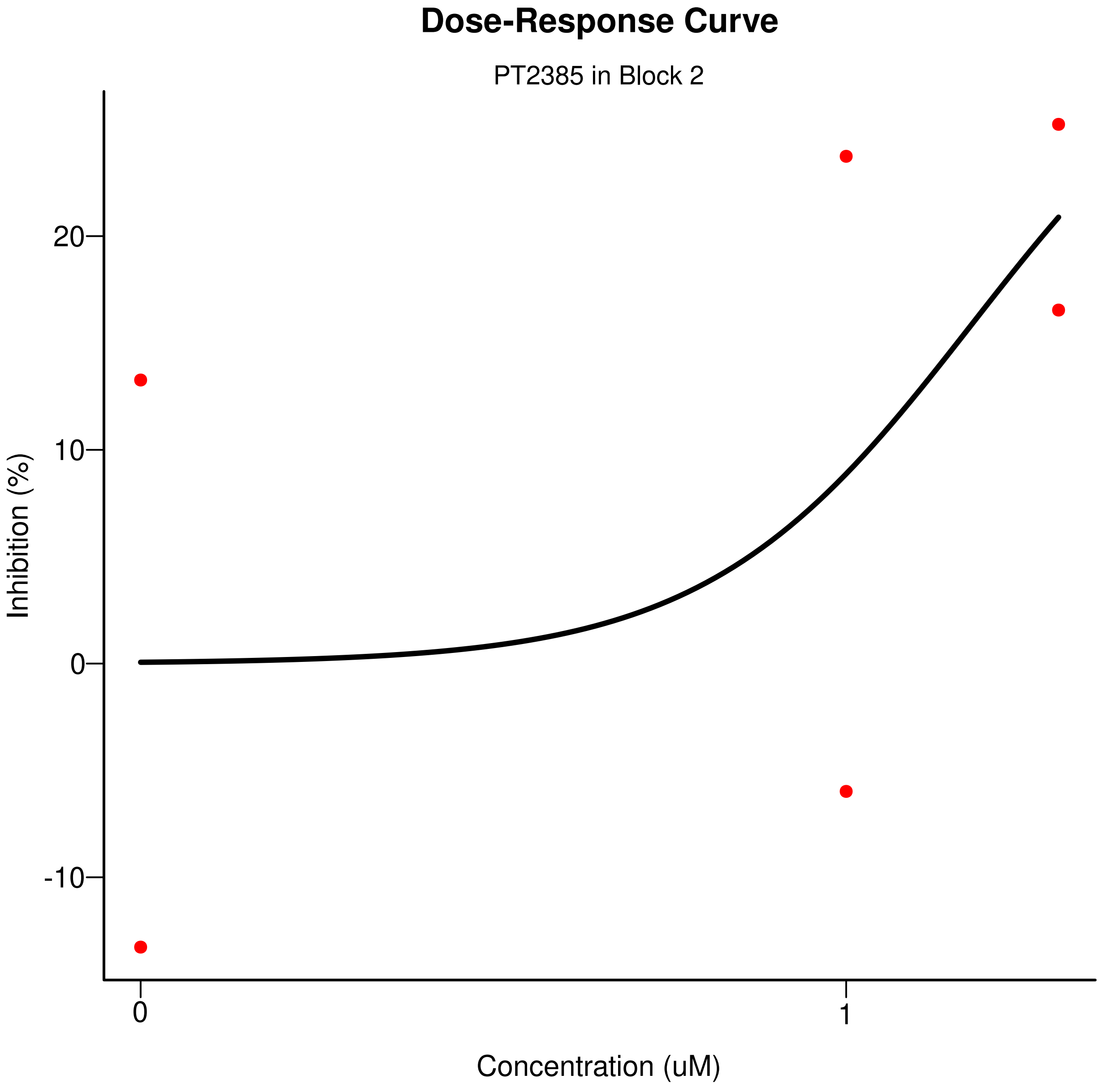

Dose-Response Curve
PT2385 in Block 2
Inhibition (%)
20
10
0
-10
0
1
Concentration (uM)

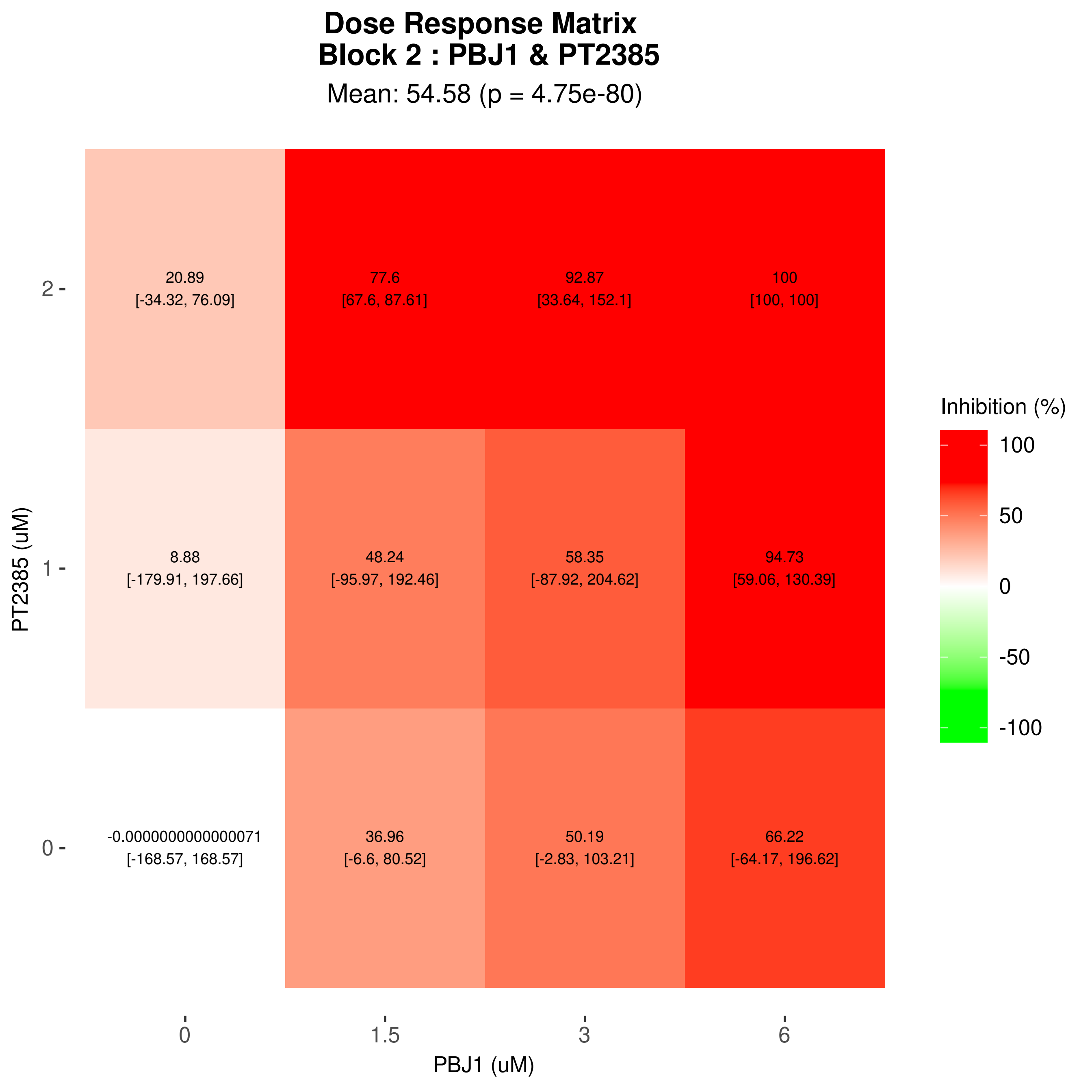
Dose Response Matrix
Block 2 : PBJ1 & PT2385
Mean: 54.58 (p = 4.75e-80)
20.89
[-34.32, 76.09]
77.6
[67.6, 87.61]
92.87
[33.64, 152.1]
100
[100, 100]
8.88
[-179.91, 197.66]
48.24
[-95.97, 192.46]
58.35
[-87.92, 204.62]
94.73
[59.06, 130.39]
-0.0000000000000071
[-168.57, 168.57]
36.96
[-6.6, 80.52]
50.19
[-2.83, 103.21]
66.22
[-64.17, 196.62]
PT2385 (uM)
2
1
0
PBJ1 (uM)
0
1.5
3
6
Inhibition (%)
100
50
0
-50
-100

ZIP Synergy Score
Block 2 : PBJ1 & PT2385

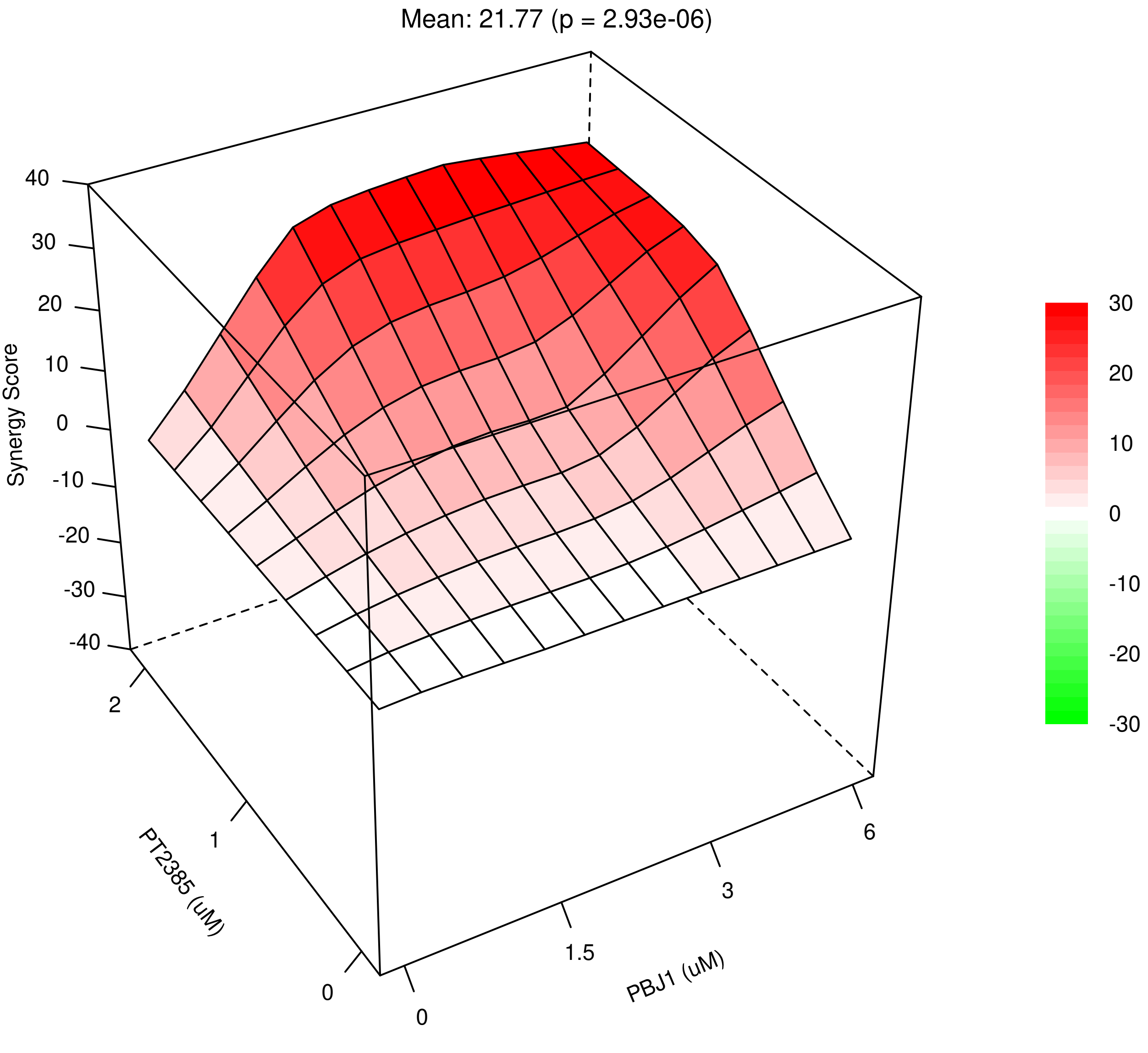

Loewe Synergy Score
Block 2 : PBJ1 & PT2385

Mean: 27.56 (p = 8.16e-18)

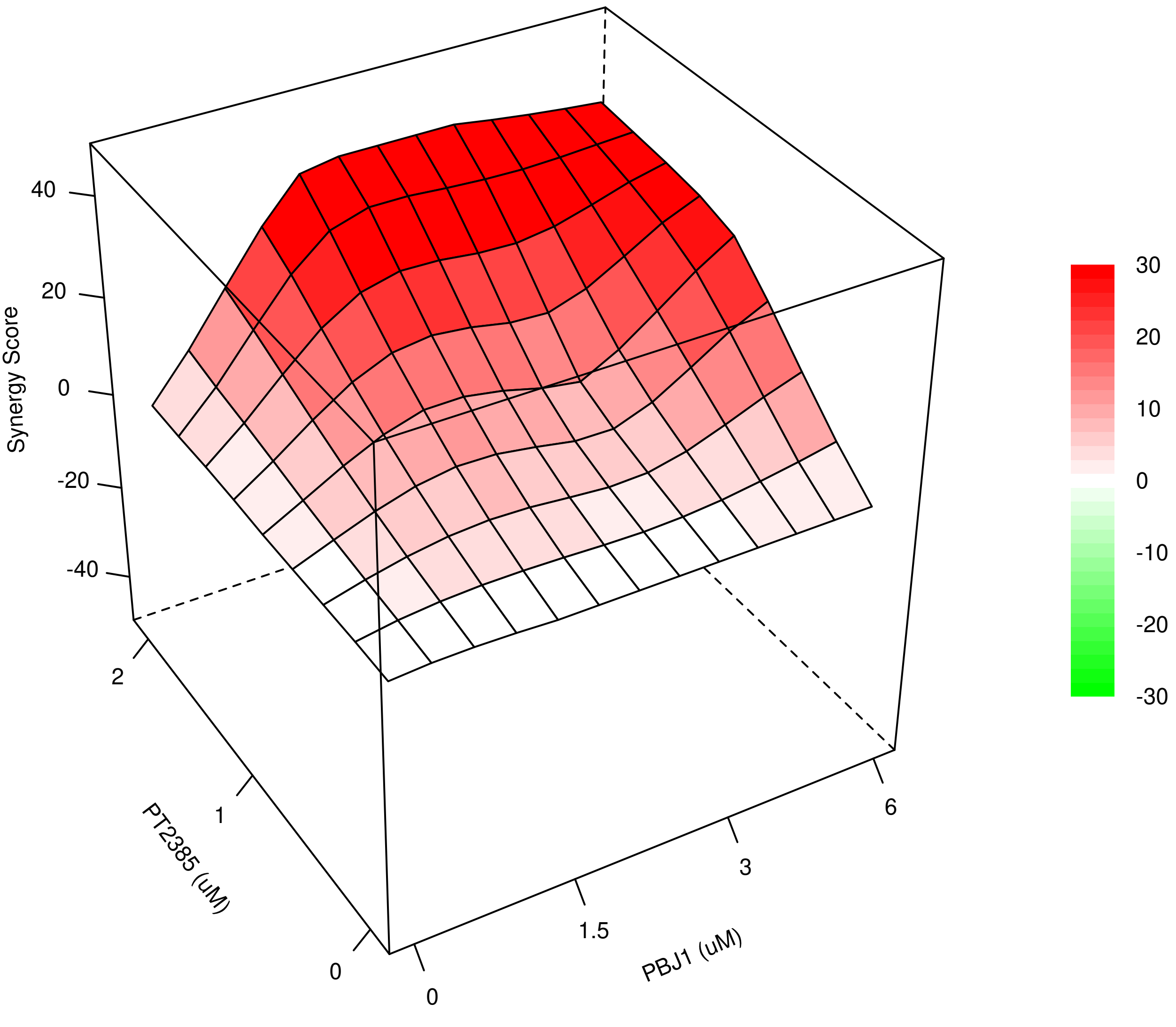

Bliss Synergy Score
Block 2 : PBJ1 & PT2385

Mean: 20.45 (p = 5.30e-04)

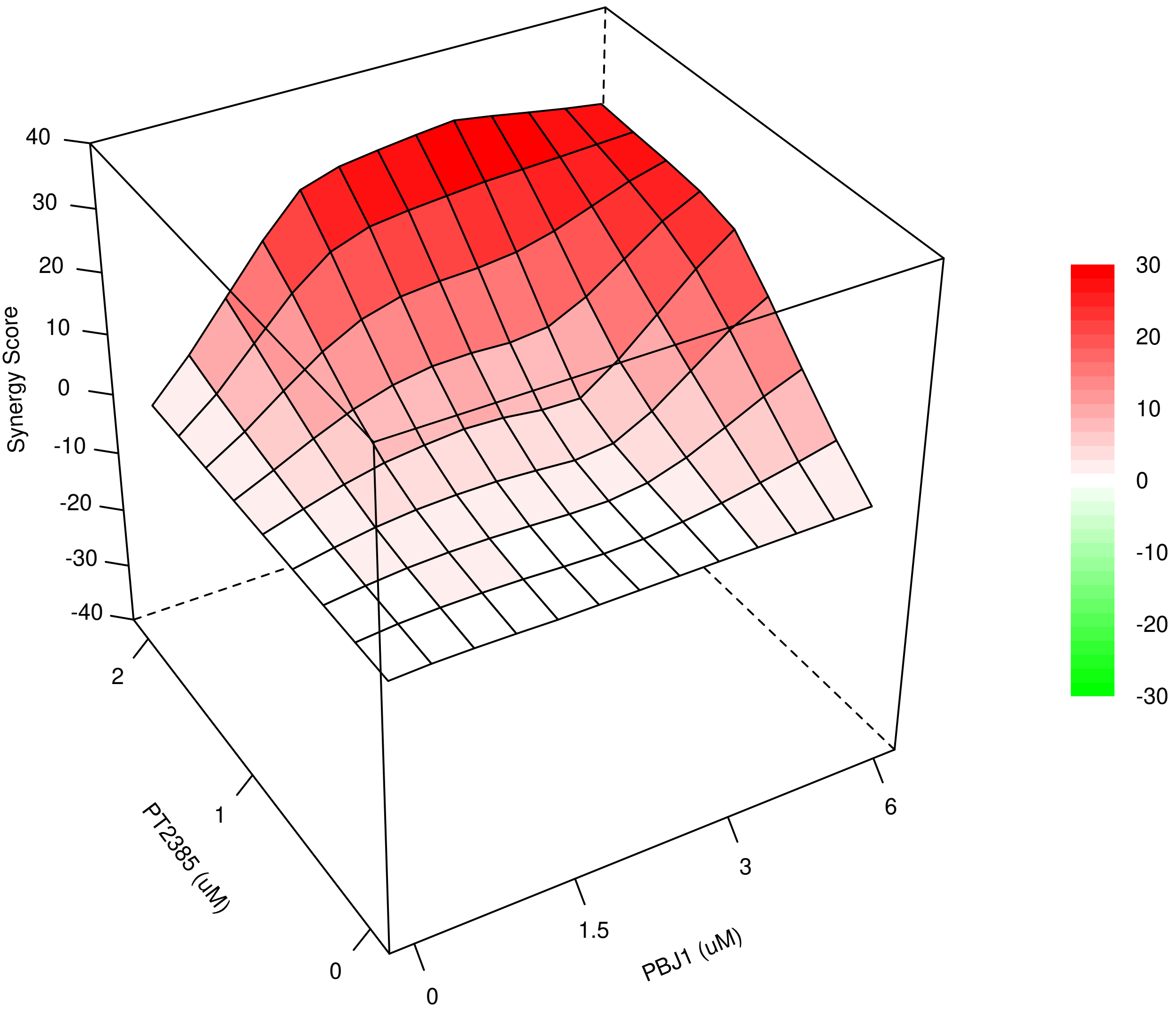

HSA Synergy Score
Block 2 : PBJ1 & PT2385

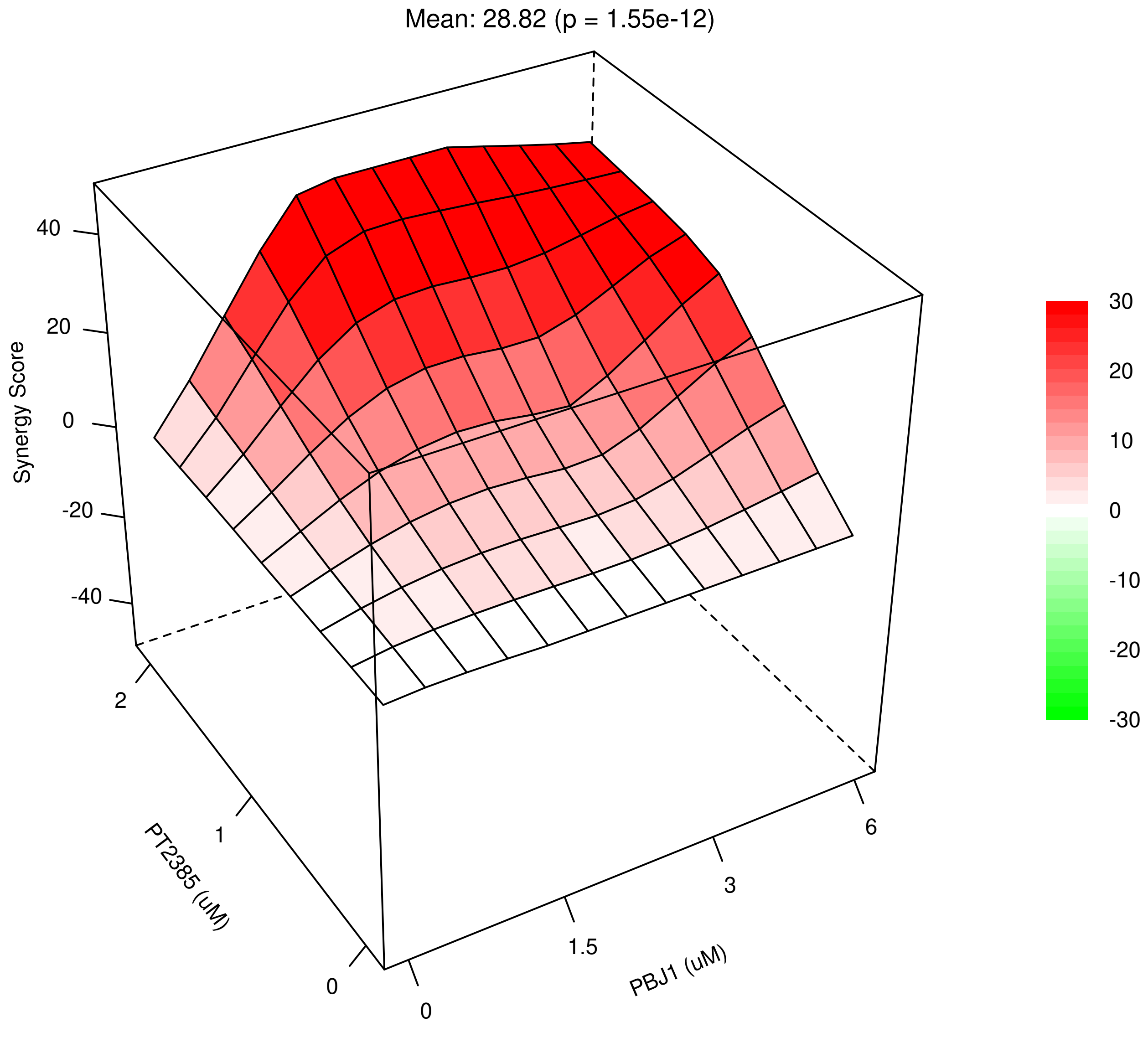

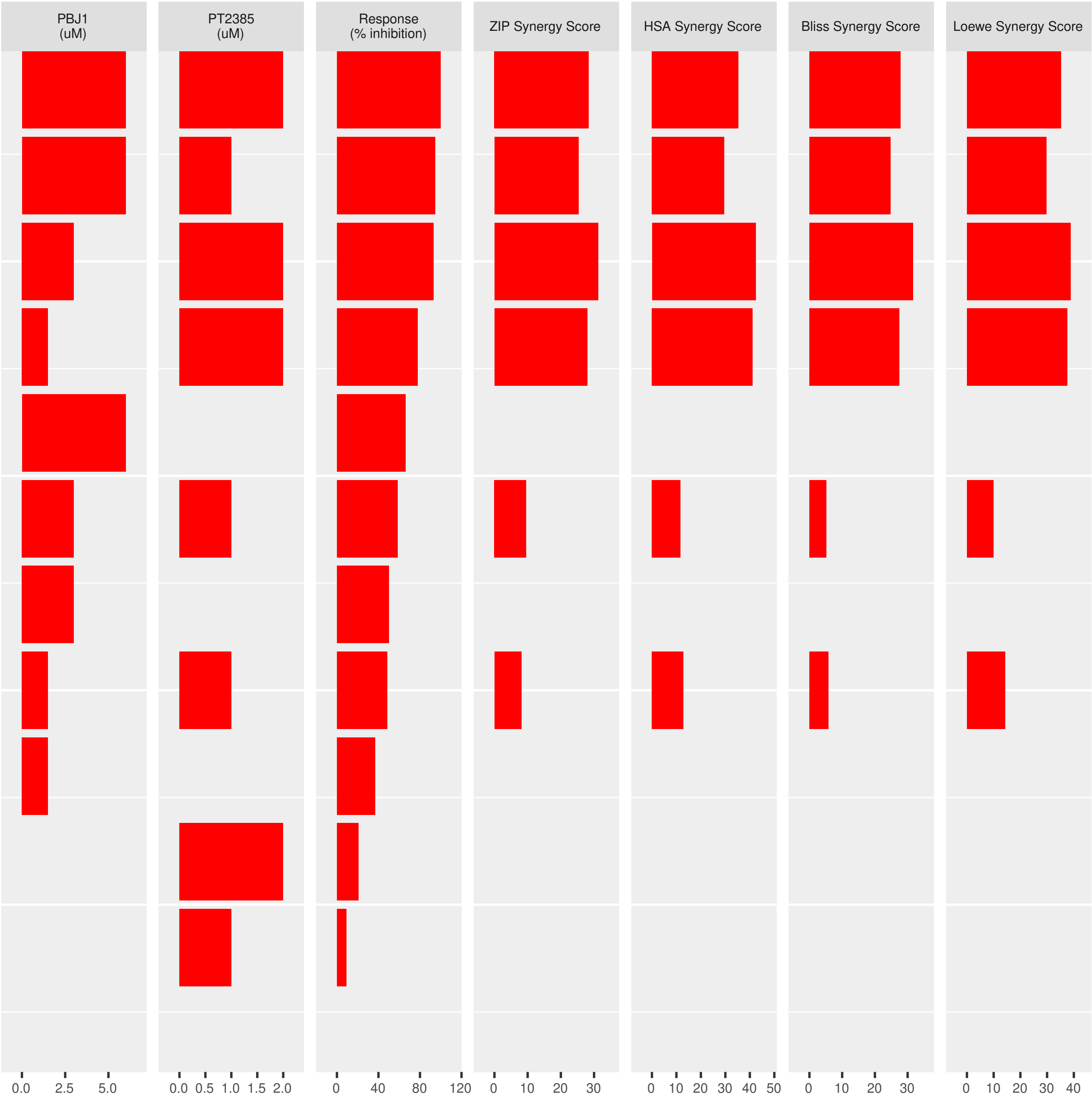
PBJ1
(uM)
PT2385
(uM)
Response
(% inhibition)
ZIP Synergy Score
HSA Synergy Score
Bliss Synergy Score
Loewe Synergy Score
0.0
2.5
5.0
0.0
0.5
1.0
1.5
2.0
0
40
80
120
0
10
20
30
0
10
20
30
40
50
0
10
20
30
0
10
20
30
40

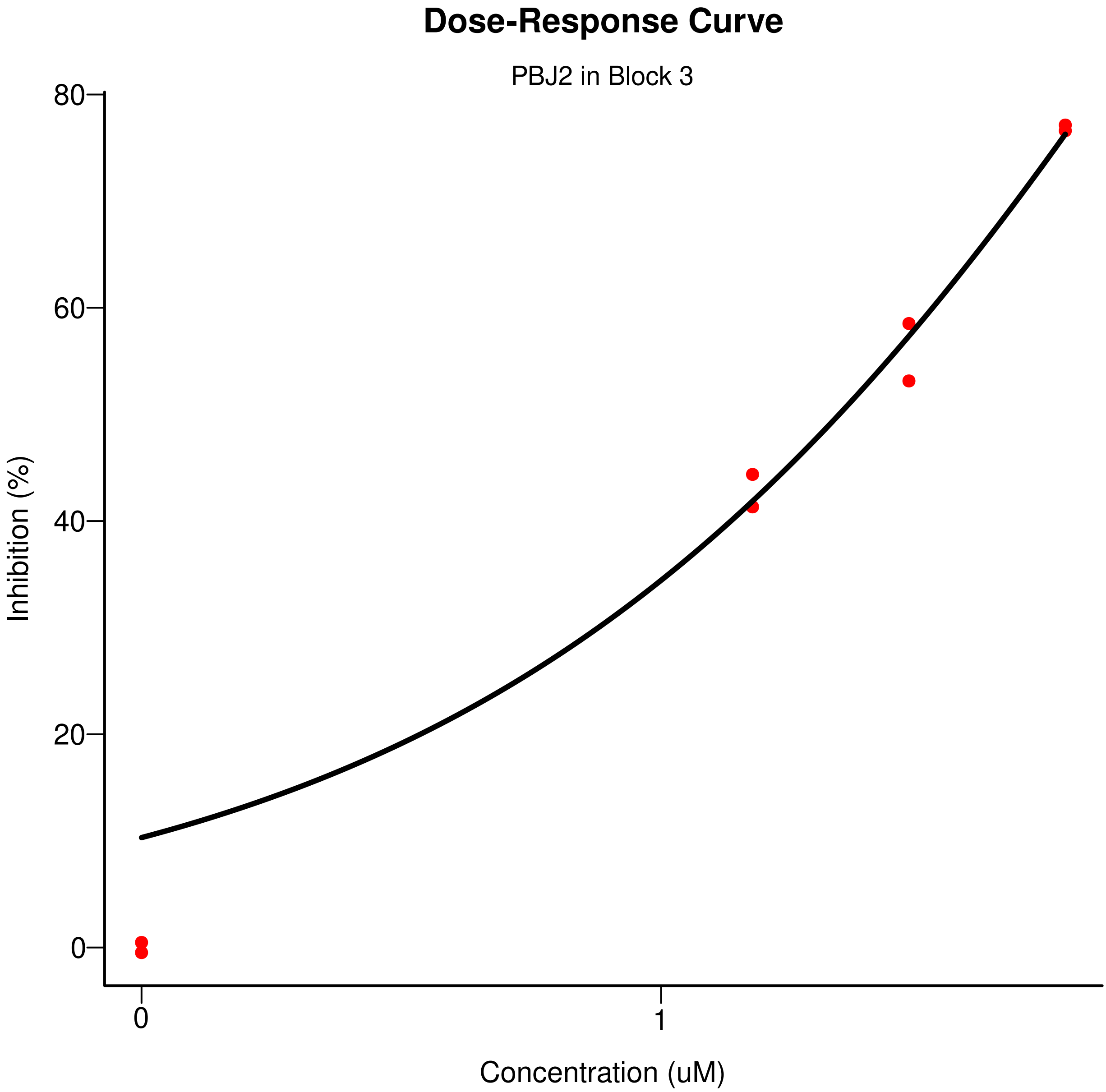

Dose-Response Curve
PBJ2 in Block 3
80
60
40
20
0
Inhibition (%)
0
1
Concentration (uM)

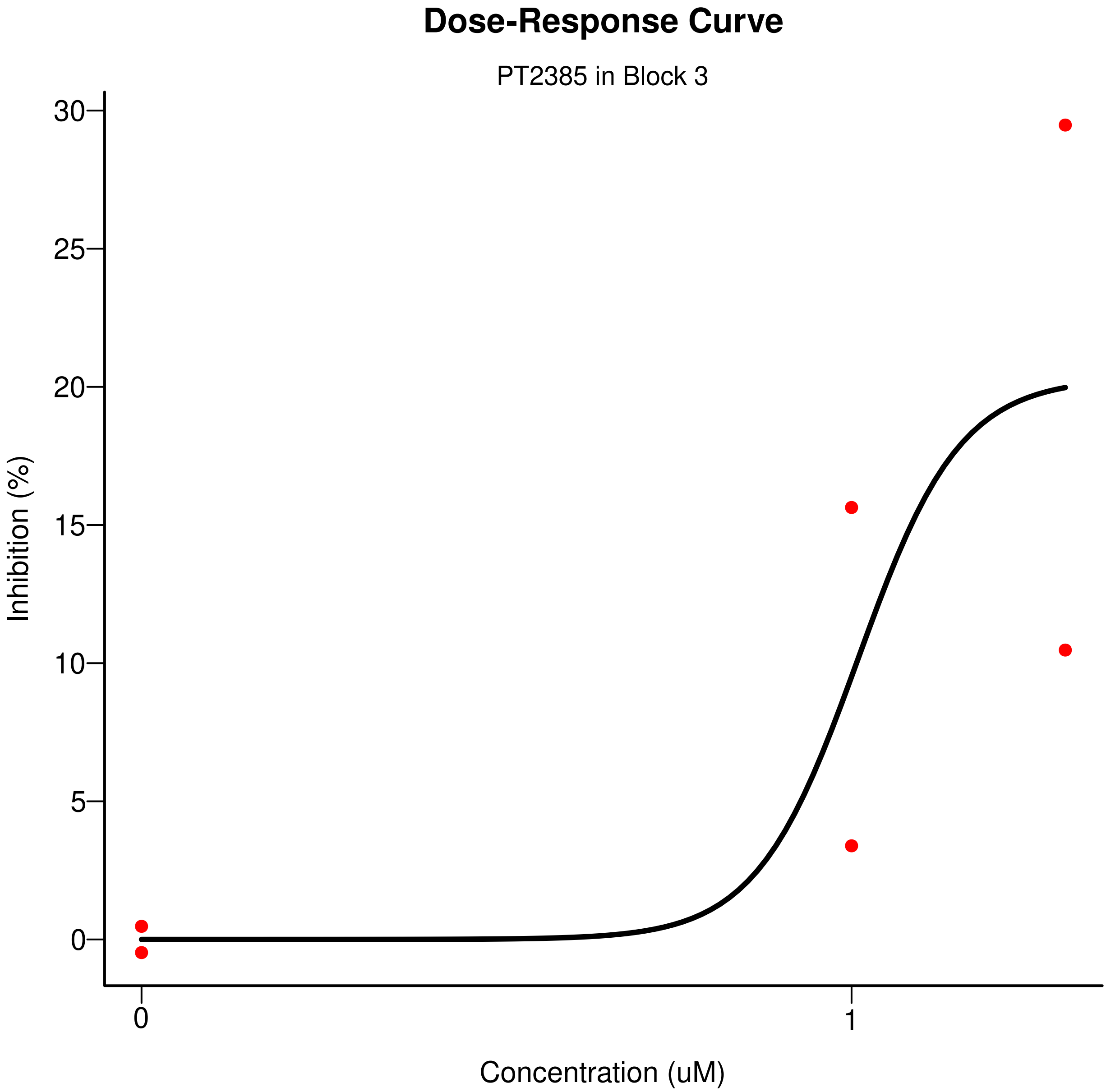

Dose-Response Curve
PT2385 in Block 3
Inhibition (%)
30
25
20
15
10
5
0
0
1
Concentration (uM)

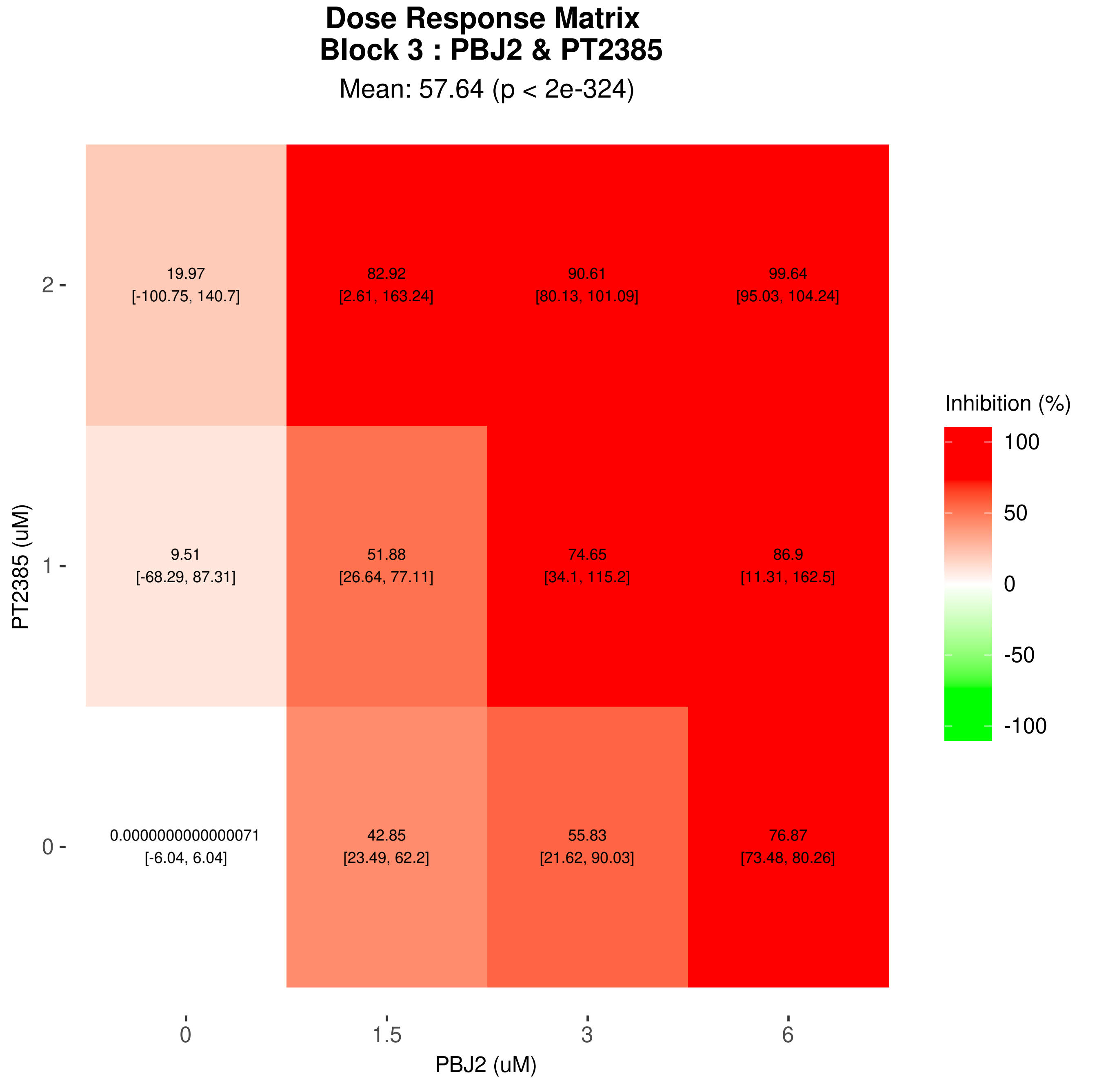
Dose Response Matrix
Block 3 : PBJ2 & PT2385
Mean: 57.64 (p < 2e-324)
PT2385 (uM)
PBJ2 (uM)
Inhibition (%)
100
50
0
-50
-100
2
1
0
0
1.5
3
6
19.97
[-100.75, 140.7]
82.92
[2.61, 163.24]
90.61
[80.13, 101.09]
99.64
[95.03, 104.24]
9.51
[-68.29, 87.31]
51.88
[26.64, 77.11]
74.65
[34.1, 115.2]
86.9
[11.31, 162.5]
0.0000000000000071
[-6.04, 6.04]
42.85
[23.49, 62.2]
55.83
[21.62, 90.03]
76.87
[73.48, 80.26]

ZIP Synergy Score
Block 3 : PBJ2 & PT2385

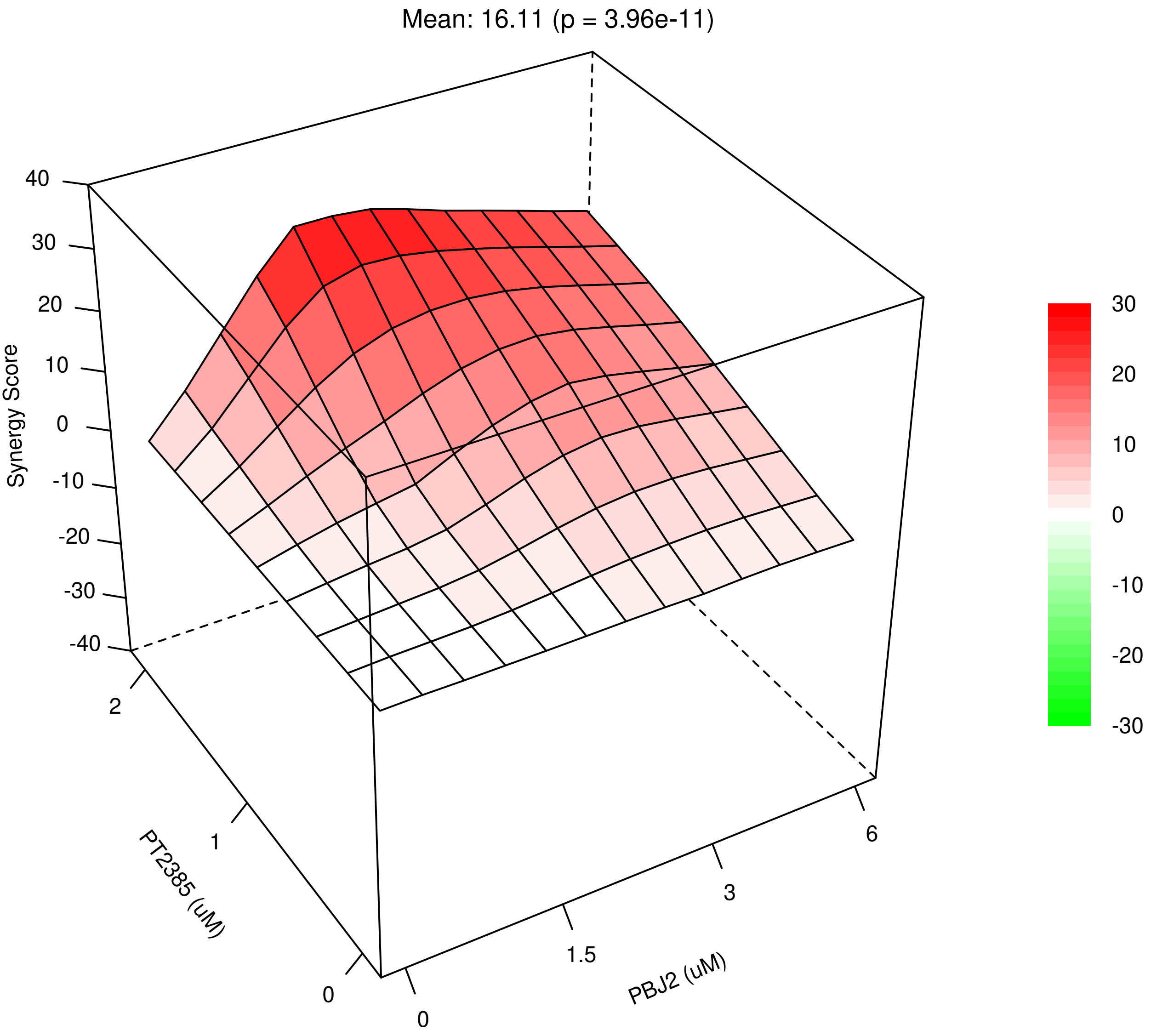

Loewe Synergy Score
Block 3 : PBJ2 & PT2385

Mean: 22.58 (p = 2.43e-43)

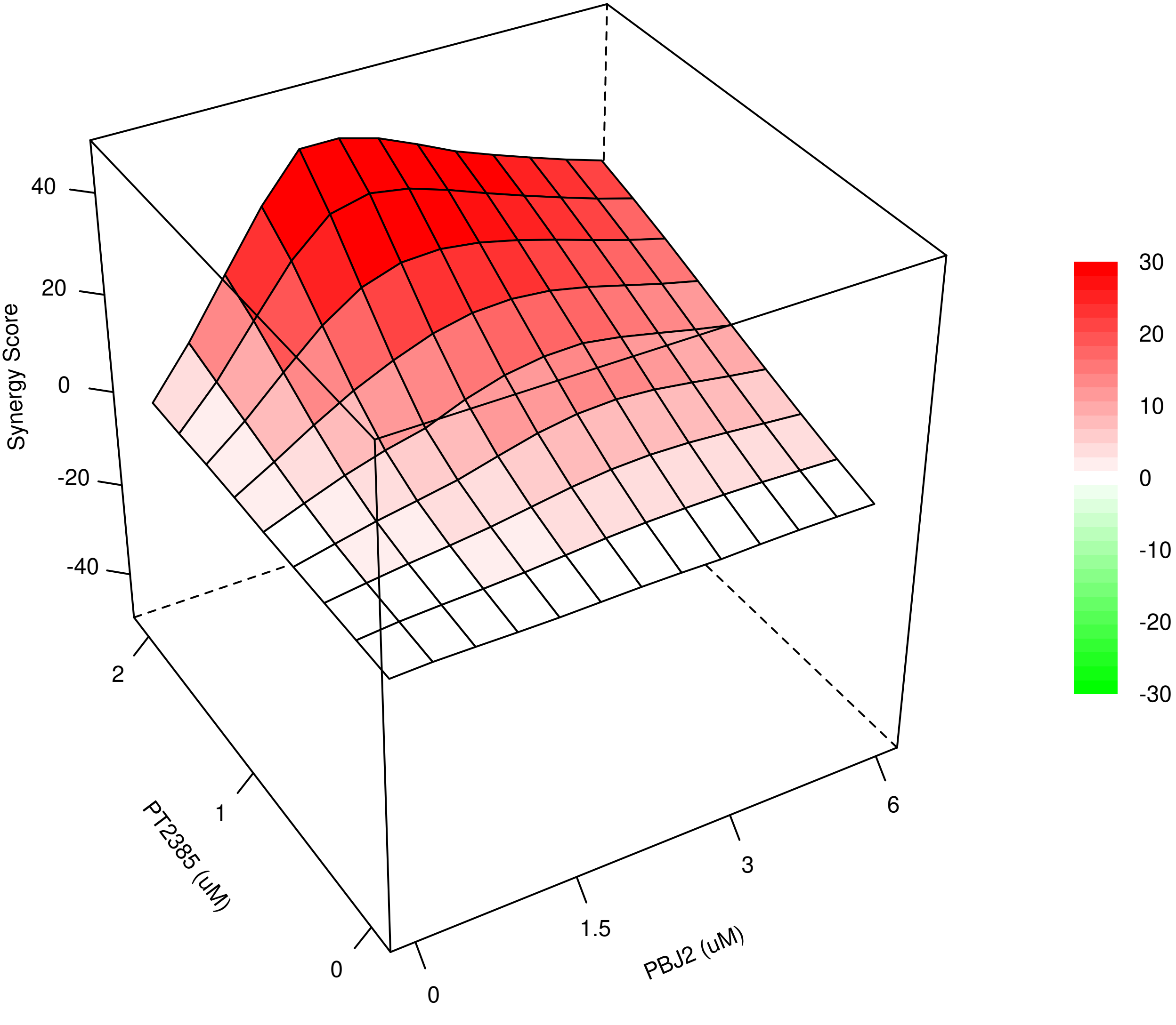

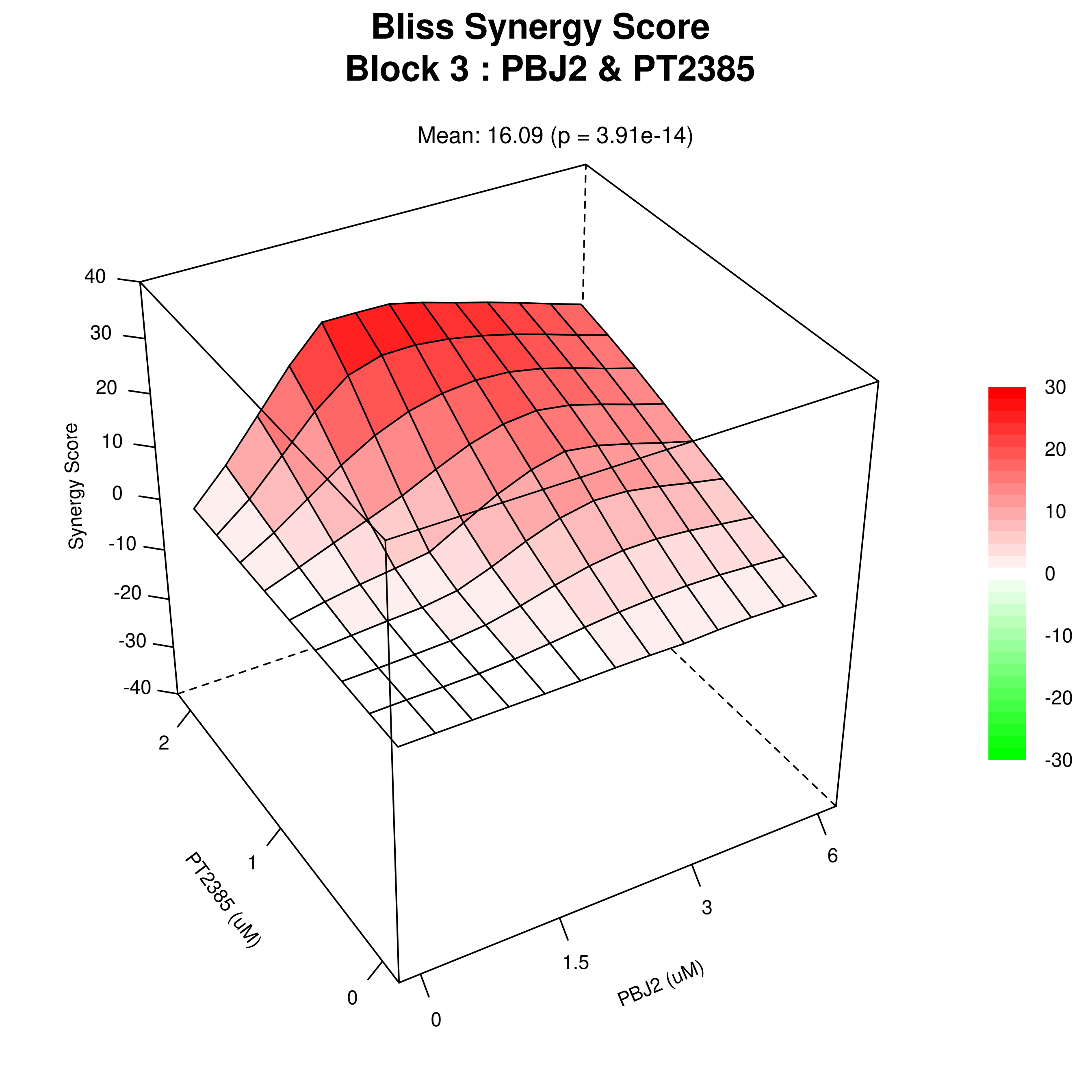

Bliss Synergy Score
Block 3 : PBJ2 & PT2385
Mean: 16.09 (p = 3.91e-14)
Synergy Score
40
30
20
10
0
-10
-20
-30
-40
PT2385 (uM)
2
1
0
PBJ2 (uM)
0
1.5
3
6
30
20
10
0
-10
-20
-30

HSA Synergy Score
Block 3 : PBJ2 & PT2385

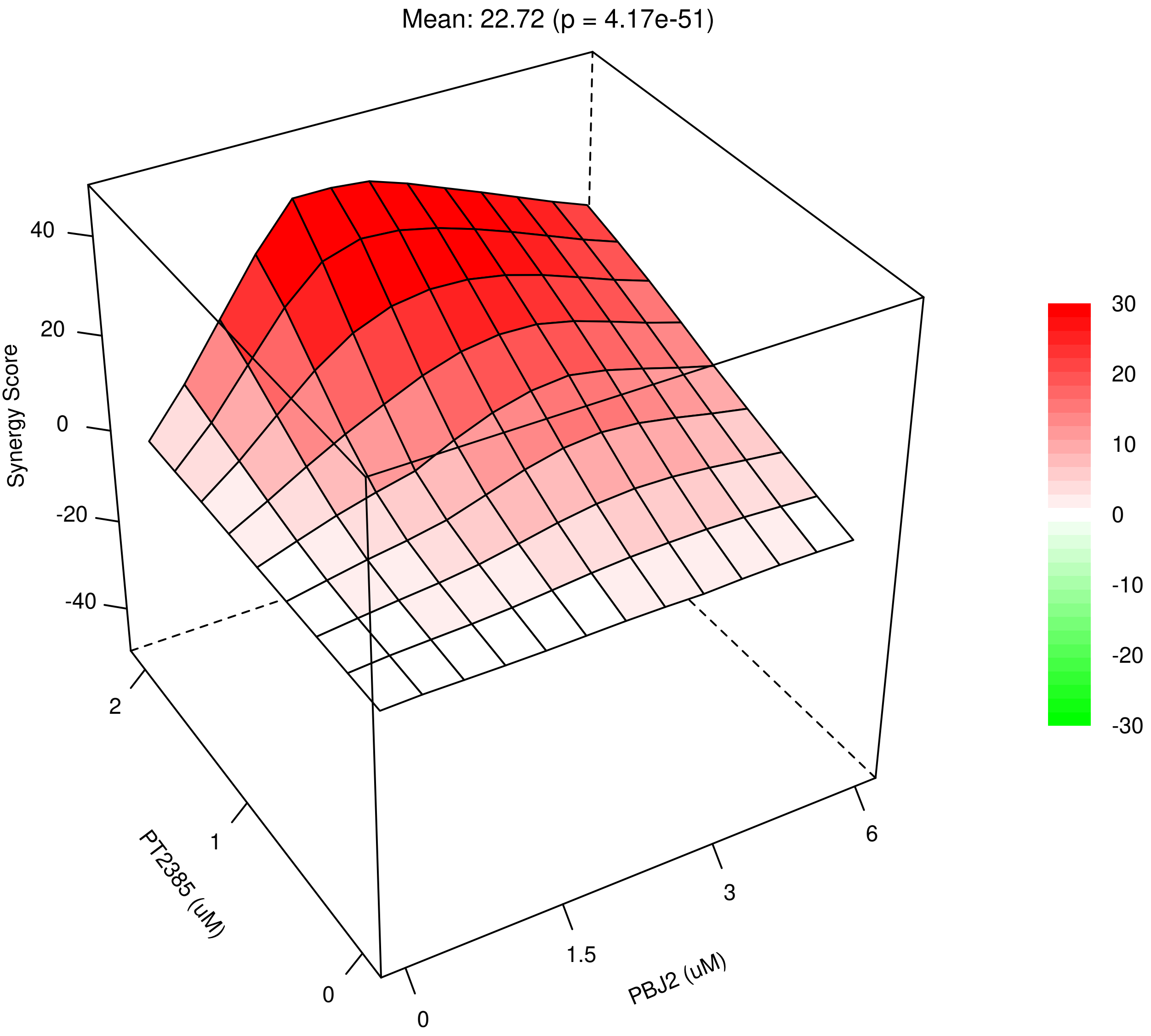

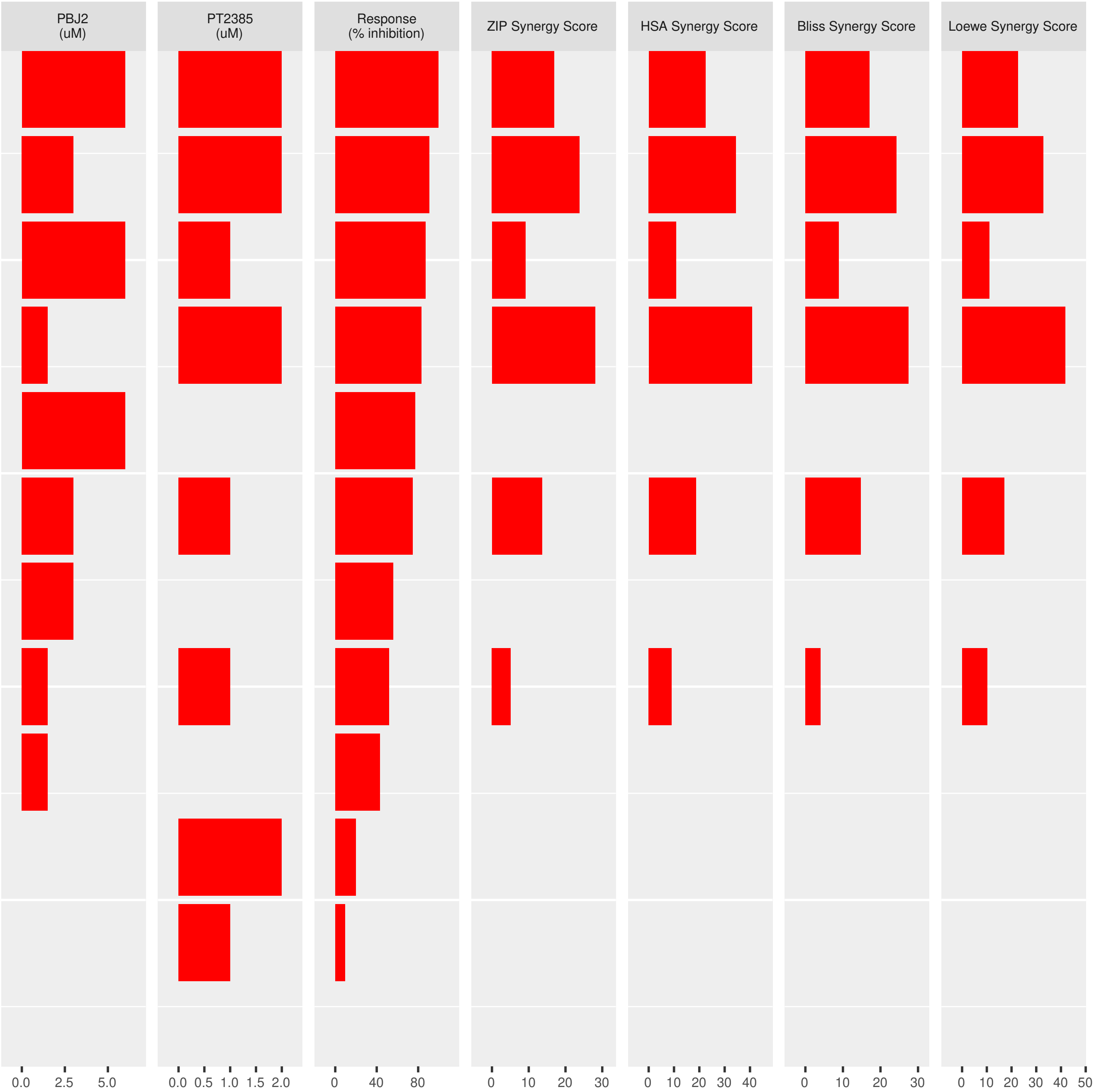

PBJ2 (uM)
PT2385 (uM)
Response (% inhibition)
ZIP Synergy Score
HSA Synergy Score
Bliss Synergy Score
Loewe Synergy Score
0.0 2.5 5.0
0.0 0.5 1.0 1.5 2.0
0 40 80
0 10 20 30
0 10 20 30 40
0 10 20 30
0 10 20 30 40 50
