# Supplementary material for: Structure-guided optimization of SLC1A1/EAAT3-selective inhibitors targeting renal cancer metabolism
Source: EMBO J. 2026 Apr 22;45(11):3763–87. doi: 10.1038/s44318-026-00776-2 (PMC13226657; doi:10.1038/s44318-026-00776-2)
Supplement: Supplementary file 6 — Source data Fig. 5 [file 44318_2026_776_MOESM6_ESM.zip › Figure 5/5I-K/SynergyFinder_report_2025-12-10.zip!/data_tables.pdf]

SynergyFinder+ Report

https://synergyfinder.org/ http://synergyfinder.ai/ www.synergyfinderplus.org
https://tangsoftwarelab.shinyapps.io/synergyfinder

2025-12-10

```
## Correct Baseline Setting:  non
```

Data tables

Table 1: Drug Combiantion Meta Data

Block ID	$Drug_1$	$Drug_2$	Conc $Unit_1$	Conc $Unit_2$
1	3e	PT2385	uM	uM
2	PBJ1	PT2385	uM	uM
3	PBJ2	PT2385	uM	uM

Table 2: Synergy Score Summary Table

Block ID	ZIP	Loewe	HSA	Bliss
1	2.62	10.83	12.87	3.87
2	21.77	27.56	28.82	20.45
3	16.11	22.58	22.72	16.09

Table 3: Synergy Score Statistic Table

Block ID	ZIP p Value	Loewe p Value	HSA p Value	Bliss p Value
1	5.20e-01	1.34e-03	4.12e-05	3.34e-01
2	2.93e-06	8.16e-18	1.55e-12	5.30e-04
3	3.96e-11	2.43e-43	4.17e-51	3.91e-14

Reference

For use of the SynergyFinder+ R package or the web application:

[1] Zheng, S.; Wang, W.; Aldahdooh, J.; Malyutina, A.; Shadbahr, T.; Tanoli, Z.; Passia, A.; Tang, J. SynergyFinder Plus: Toward Better Interpretation and Annotation of Drug Combination Screening Datasets. Genomics, Proteomics & Bioinformatics 2022, 20 (3), 587-596. doi:10.1016/j.gpb.2022.01.004.

For use of ZIP synergy scoring:

[2] Yadav, B.; Wennerberg, K.; Aittokallio, T.; Tang, J. Searching for Drug Synergy in Complex Dose-Response Landscapes Using an Interaction Potency Model. Comput Struct Biotechnol J 2015, 13, 504–513. doi:10.1016/j.csbj.2015.09.001

For how to harmonize the different synergy scoring methods:

[3] Tang, J.; Wennerberg, K.; Aittokallio, T. What Is Synergy? The Saariselkä Agreement Revisited. Front Pharmacol 2015, 6, 181. doi:10.3389/fphar.2015.00181

For general ideas of drug combination therapies:

[4] Tang, J. Informatics Approaches for Predicting, Understanding, and Testing Cancer Drug Combinations. Methods Mol Biol 2017, 1636, 485–506. doi:10.1007/978-1-4939-7154-1_30

For retrieving the most comprehensive drug combination data resources and their sensitivity and synergy results by SynergyFinder, please go to DrugComb :

[5] Zheng, S.; Aldahdooh, J.; Shadbahr, T.; Wang, Y.; Aldahdooh, D.; Bao, J.; Wang, W.; Jing, T. DrugComb update: a more comprehensive drug sensitivity data repository and analysis portal. Nucleic Acids Research 2021, 49 (w1), w174-w184. doi:10.1093/nar/gkab438

[6] Zagidullin, B.; Aldahdooh, J.; Zheng, S.; Wang, W.; Wang, Y.; Saad, J.; Malyutina, A.; Jafari, M.; Tanoli, Z.; Pessia, A.; Tang, J. DrugComb: An Integrative Cancer Drug Combination Data Portal. Nucleic Acids Res 2019, 47 (W1), W43–W51. doi:10.1093/nar/gkz337

For use of combination sensitivity score:

[7] Malyutina, A.; Majumder, M. M.; Wang, W.; Pessia, A.; Heckman, C. A.; Tang, J. Drug Combination Sensitivity Scoring Facilitates the Discovery of Synergistic and Efficacious Drug Combinations in Cancer. PLOS Computational Biology 2019, 15 (5), e1006752. doi:10.1371/journal.pcbi.1006752
